# Female proportion has a stronger influence on dispersal than body size in nematodes of mountain lakes

**DOI:** 10.1371/journal.pone.0303864

**Published:** 2024-05-17

**Authors:** Guillermo de Mendoza, Birgit Gansfort, Jordi Catalan, Walter Traunspurger

**Affiliations:** 1 Institute of Geography, Faculty of Oceanography and Geography, University of Gdansk, Gdańsk, Poland; 2 Institute of Biology and Earth Sciences, Pomeranian University in Słupsk, Słupsk, Poland; 3 Department of Animal Ecology, Bielefeld University, Bielefeld, Germany; 4 CREAF, Cerdanyola del Vallès, Barcelona, Spain; 5 CSIC, Bellaterra, Barcelona, Spain; University of Uyo, NIGERIA

## Abstract

Nematodes disperse passively and are amongst the smallest invertebrates on Earth. Free-living nematodes in mountain lakes are highly tolerant of environmental variations and are thus excellent model organisms in dispersal studies, since species-environment relationships are unlikely to interfere. In this study, we investigated how population or organism traits influence the stochastic physical nature of passive dispersal in a topologically complex environment. Specifically, we analyzed the influence of female proportion and body size on the geographical distribution of nematode species in the mountain lakes of the Pyrenees. We hypothesized that dispersal is facilitated by (i) a smaller body size, which would increase the rate of wind transport, and (ii) a higher female proportion within a population, which could increase colonization success because many nematode species are capable of parthenogenetic reproduction. The results showed that nematode species with a low proportion of females tend to have clustered spatial distributions that are not associated with patchy environmental conditions, suggesting greater barriers to dispersal. When all species were pooled, the overall proportion of females tended to increase at the highest elevations, where dispersal between lakes is arguably more difficult. The influence of body size was barely relevant for nematode distributions. Our study highlights the relevance of female proportion as a mechanism that enhances the dispersal success of parthenogenetic species, and that female sex is a determining factor in metacommunity connectivity.

## Introduction

A fundamental goal in community ecology is to decipher the roles played by spatial and environmental factors in shaping species distributions and community assembly when the regional species pool affects local community dynamics [1–4]. Using a multi-scale approach has led to the concept of metacommunity, defined as a set of more or less isolated local communities potentially connected by dispersal [1]. This framework has been increasingly applied to understand the relative importance of the spatial (i.e., dispersal-related), environmental (i.e., niche-based), and stochastic processes structuring communities [5–7]. Typical metacommunity analyses have investigated the amount of variation in community similarity among sites that is explained by environmental and spatial variables. Their findings have revealed a context-dependency imposed by environmental conditions or the influence of the dispersal traits of the organismal groups under consideration (e.g., [8, 9]). Another approach has been to investigate single-species distributions and then compare the results across species in a meta-analysis. This approach has also evidenced the relevant roles of environmental context and dispersal traits in species distributions (e.g., [10]).

**Fig 1:**
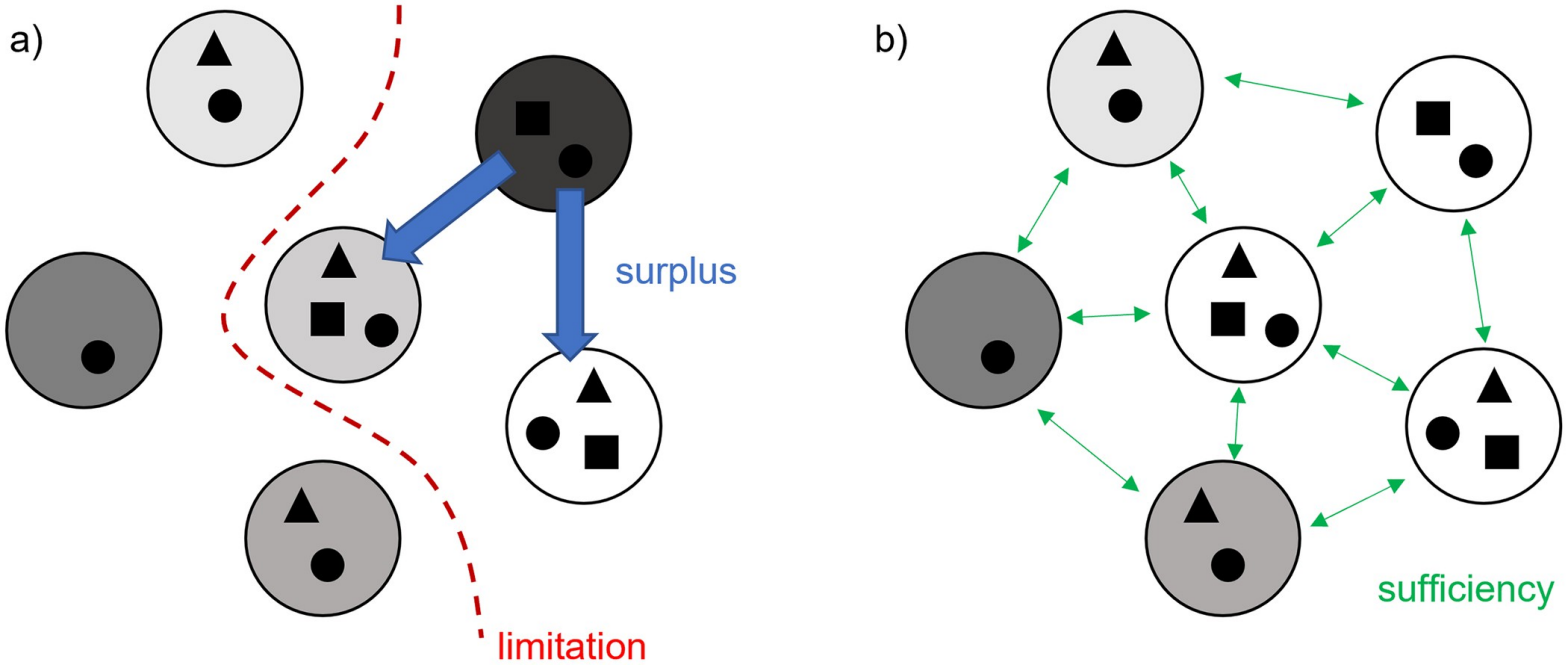
Schematic metacommunity scenarios depicting the role of dispersal in the spatial structuring of metacommunities: Black squares, circles, and triangles indicate different species; the large white circles and the different shades of gray indicate (environmentally heterogeneous) local communities. The squares may be spatially clustered (a) due to dispersal limitation and/or dispersal surplus and (b) due to the spatial arrangement of environmental conditions even if dispersal is sufficient.

The connectivity between local sites reflects both dispersal strength and environmental barriers [4, 11]. Under dispersal limitation, species cannot reach every site where they might otherwise persist, resulting in spatial metacommunity structuring, while in the absence of dispersal barriers, the amount of dispersal will be sufficient for a species to track environmental gradients and establish populations at any suitable site. In the latter, patchy or spatially autocorrelated environmental conditions may explain metacommunity structuring. A third scenario occurs when high dispersal rates between neighboring sites cause a species at a given location to persist under suboptimal conditions, due to the constant immigration of individuals from neighboring optimal sites, which act as a constant input source (i.e., mass effects or source-sink dynamics, see [1]). These three scenarios can be referred to as *dispersal limitation*, *dispersal sufficiency*, and *dispersal surplus*, respectively (as described in the book of Leibold and Chase [4]; Fig 1). Dispersal limitation can produce spatially structured metacommunities at large spatial scales while dispersal surplus can be expected among closely located sites and should therefore generate small-scale spatial signals [11]. In the case of dispersal sufficiency, spatially autocorrelated environmental conditions are likely to be the main cause of spatial structuring. However, although the theoretical basis for distinguishing potential causes of metacommunity structuring may be intuitive, determining whether the observed pattern is due to spatially structured environmental conditions or dispersal-related processes can be challenging in practice [5, 12].

Species traits, such as dispersal mode (passive vs. active) or body size, may influence dispersal potential. Previous studies have found that organisms with a low dispersal ability have a stronger spatial structure in their distributions than those able to disperse over larger distances and/or at higher dispersal rates [9, 13, 14]. In freshwater habitats, it has been suggested that actively-dispersing insects with flying adults more closely track favorable environmental conditions than passive dispersers [12, 15–17]. Within the latter, however, spatial effects on species distributions appear stronger in organisms with larger propagules [8, 15], in line with the positive influence of small body size on the passive dispersal potential of a species [18]. In the study of Ptatscheck et al. [19], the proportion of small nematodes in the aeroplankton caught far away from potential home habitats was larger than in samples collected close to emigration sources, suggesting that larger nematode species are more prone to dispersal limitation. In addition to body size and dispersal mode, species traits such as the presence of resting stages, population size, and fecundity may significantly influence the dispersal efficiency of aquatic invertebrates [20, 21].

Nematodes are ubiquitous. They are amongst the smallest invertebrates on Earth, disperse passively, and many species can establish new populations without males, by reproducing parthenogenetically or as hermaphrodites. Moreover, gravid females can establish populations without invoking parthenogenesis or hermaphroditism. Accordingly, successful colonization does not require that females encounter a male at the same site, such that females may be more valuable than males in establishing populations at new sites. Also, nematodes are usually the most dominant group of meiobenthic organisms in terms of abundance and species richness [22–24]. In addition, many species are highly tolerant of environmental variation [16, 25], which increases the number of suitable sites to be colonized. Nematodes are therefore good models for testing the influence of species traits such as body size and sex ratio on species distributions and metacommunity structuring [21, 26]. Their use may avoid the strong influence of species-environmental relationships typical of many organismal groups. Nonetheless, nematode-based metacommunity studies are mostly lacking [26], although the few studies analyzing the drivers of nematode metacommunities have demonstrated that spatial structuring is a common characteristic [17, 27, 28].

In this study, we explored the influence of female dominance and body size on the geographical distribution (i.e., latitude and longitude) of nematode species in the mountain lakes of the Pyrenees and how these two traits vary between lakes across the elevational gradient. Our working hypothesis was that a higher female dominance and smaller body size increase the dispersal potential. We tested our hypothesis and its implications by using single-species models to evaluate the role of environmental and spatial variables on 2-dimensional geographical distributions, with spatial variables considered at small, medium, and large scales. We predicted that a lower proportion of females and a larger body size would relate to the ability of spatial variables to explain distributions after the effect of environmental variables is partialled out. Large-scale spatial variables representing the large autocorrelation structures should be expected to explain nematode distributions under a *dispersal limitation* scenario, while small-scale spatial variables should be expected to do so under a *dispersal surplus* scenario. As a complementary analysis, we calculated Moran’s autocorrelation index (*I*) for each species to estimate its degree of clustering in space. Higher *I* values were expected for nematode species with a lower proportion of females and larger body sizes. We also checked the potential influence of nematode’s species abundance (as a surrogate of density) on the ability of the different subsets of variables to explain their distributions, as well as the potential effect of abundance on Moran’s *I*. Finally, concerning the altitudinal gradient, a higher dominance of females and nematode species of smaller size were expected at lakes located at the highest elevations where dispersal is arguably more difficult since the availability of suitable habitats becomes reduced and more distant apart at the highest elevations.

## Material and methods

### Sampling procedure

**Fig 2:**
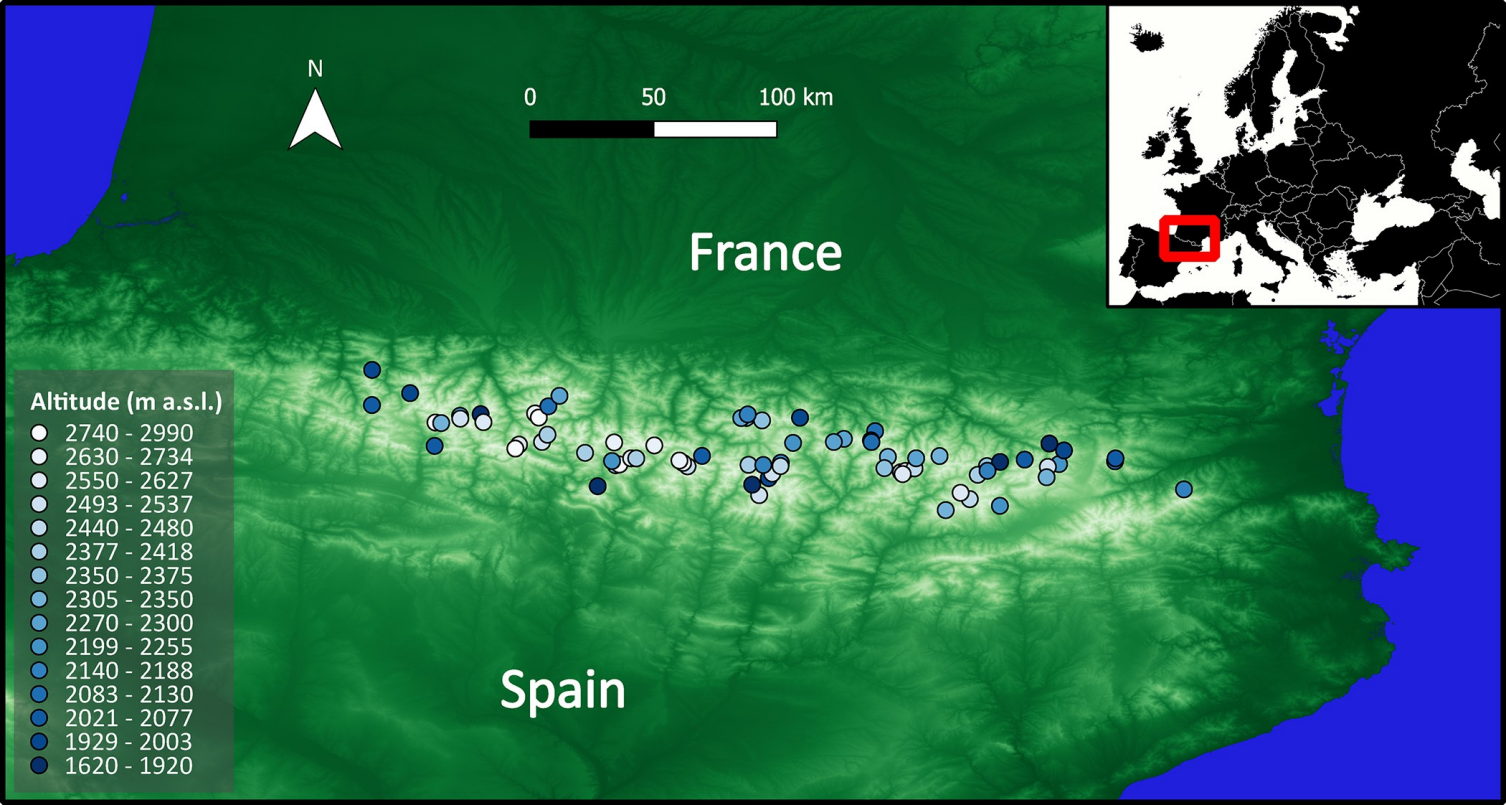
Map showing the location and altitude of the surveyed lakes in the Pyrenees where nematodes were found (n = 75). Circles represent lakes, filled with colors according to their altitude. Digital elevation model built with QGIS Desktop 3.16.13 software with elevation data from NASA (https://www.earthdata.nasa.gov). Europe map from MapChart (https://www.mapchart.net).

In the summer of 2000, 82 lakes in the Pyrenees were sampled following standardized protocols for the littoral collection of invertebrate samples in mountain areas. Permits were obtained from all the existing protected areas in the Pyrenees at that time, namely, Parc Nacional d’Aigüestortes I Estany de Sant Maurici, Parque Nacional de Ordesa y Monte Perdido, Parc National des Pyrénées, Parc Natural del Cadí-Moixeró, and Parque Natural de Posets-Maladeta. Nematodes were found in 75 of these lakes [16, 29]. The selected lakes represent the altitudinal and lithological variability of the lakes of the Pyrenees and thus cover most of the physical and chemical variation of lakes in the region [30–33]. Lakes located at geographical extremes were included, making up a maximal geographical (ellipsoid) distance between lakes of 262.5 km, approximately 40 km of maximum perpendicular distance, and comprising an altitudinal range of 1370 m (i.e., from 1620 to 2990 m a.s.l.). Sampling was performed at five 1-m^2^ sampling points in the littoral zone of each lake following the kick-sampling method. A hand net with a 100-μm mesh was used in the field (but a 250-μm sieve in sample processing in the lab). The sampling points at each lake were specifically selected to proportionally represent the physical and aquatic vegetation variability of the benthic habitats of the lake (estimated by an *in situ* exploration of the lake’s littoral zone by several observers independently). All samples in a given lake were later pooled to form a single sample representing 5 m^2^ of benthic habitats and 5 min of sampling effort per lake. The sampling procedure was described in detail in previous studies [16, 29]. Lakes where nematodes were found (n = 75) included the complete altitudinal range (Fig 2), with a maximal geographical (ellipsoid) distance of 246.8 km (247.9 km in Cartesian space, see ‘Spatial variables’ below), and ranged from 0.24 to 44.65 ha in size and from 0.7 to 100 m in depth.

### Spatial variables

Spatial variables were used to test the influence of spatially autocorrelated structures on species distributions. To obtain these autocorrelated structures, all 75 lakes where nematodes were found were first represented in Cartesian space using the function “geoXY” of the R package “SoDA” [34]. Euclidean distances between lakes were then computed using the function “dist” of R base software [35]. This allowed the application of principal coordinates of neighboring matrices (PCNMs) to these distances using the function “pcnm” of the R package “vegan” [36], with the maximum distance of a minimum-spanning tree connecting all lakes as the threshold distance. Of the 74 PCNMs obtained, 45 were positive and retained for further statistical analysis. Negative PCNMs were discarded because they do not represent positive spatial autocorrelations. The positive PCNMs were divided into three categories of spatial structure, large-scale, medium-scale, and small-scale, after carefully assessing the eigenvalues of all 45 positive PCNMs (S1 Fig). Large-scale PCNMs were defined as those with eigenvalue > 25000 (from PCNM-1 to PCNM-6), medium-scale PCNMs as those with eigenvalues between 1000 and 25000 (PCNM-7 to PCNM-30), and small-scale PCNMs as those with eigenvalues < 1000 (PCNM-31 to PCNM-45). The geographical distribution of the PCNM values for all 45 positive PCNMs, as represented in Cartesian space, is shown in S1 Appendix.

### Environmental variables

Environmental variables described the physical and chemical environment of the lakes, including the variability in the physical nature of their benthic habitats. The 28 environmental variables considered in this study were as follows: lake area, lake depth, conductivity, pH, total nitrogen (TN); total phosphorus (TP); dissolved organic carbon (DOC); dissolved silica; ammonium; calcium; magnesium; sodium; potassium; sulfate; nitrate; chloride; acid-neutralizing capacity (ANC); surface water temperature; organic matter in deep sediment (estimated as loss on ignition, LOI, a surrogate of the general trophic status of the lake); chlorophyll-*a* (Chl-*a*); bacteria (as carbon biomass in plankton samples); benthic habitat substrate granulometry (the mean relative abundance of rocks, stones, gravel, and fine substrates at the sampling points); mean relative abundance of macrophytes at the sampling points; and fish occurrence at each lake (classified as Salmonidae or *Phoxinus*, to refer to any *Salmo*, *Salvelinus* or *Oncorhynchus* species or to *Phoxinus* species, respectively). Details on the environmental variables considered in this study can be found in previous publications [16, 29, 37].

### Nematode data

All nematodes found in this study (ca. 4000 individuals) were mounted on slides with glycerin, sealed with paraffin. The species were determined by light microscopy (Leitz Dialux 20, magnification 400-1250x). The species list is provided in de Mendoza *et al*. [16], although for this study *Dorylaimus* cf. *stagnalis* was split into two species (*Dorylaimus stagnalis* and *Dorylaimus* sp.) following morphological inspections. Mermithidae were excluded from further statistical treatment since they were not determined to the species level. Still, their parasitic nature would have complicated comparisons of this group with the other nematode species. Only a few indeterminate individuals (n = 11) had to be excluded from further analysis.

Adult individuals of each species were identified as males or females, and the proportion of females per species was considered as follows: i) as the average proportion of females per lake (i.e., mean of proportions); and ii) as the overall proportion of females in all lakes combined (i.e., proportion of all individuals pooled). The former reflects potential variations of female proportion across lakes whereas the latter assumes a constant value for each species. The proportion of females was statistically analyzed for all nematode species represented by at least six adults.

The typical body size of each species was considered by estimating the average weight using adult morphometric measurements with the formula of Andrássy [38]:

$$B=\frac{L*a^{2}}{1.6*10^{6}}$$

where *B* is fresh weight (μg), *L* is nematode length (μm), and *a* is the greatest body diameter of the nematode species (μm). Values of *L* and *a* were the average values from our measurements and those reported in the literature [39–42].

Only those nematodes present in at least four lakes were considered for the single-species distribution models. Additionally, *Plectus cirratus* was discarded because fewer than six adults were collected. For all nematodes present in at least four lakes and represented by at least six adults, Moran’s *I* spatial autocorrelation index was calculated with the function “Moran.I” of the R package “ape” [43], using a binary matrix (i.e., lakes are assumed as directly connected, or not, by the model) with the maximum distance to the nearest lake in the minimum spanning tree connecting all lakes as the threshold distance (ca. 23.6 km). In practice, this assumes that a nematode passively traveling through the air can reach lakes that are directly connected (i.e., below the threshold distance) to the source lake; otherwise, intermediate lakes are needed in a stepwise process. The significance of the observed Moran’s *I* was tested against the null expected value, assuming normality [43].

### Single-species models

The presence/absence of each nematode species was first regressed on the environmental and spatial variables using generalized linear models (GLMs) with a binomial response and logit link, with the function “glm” of the base R software [35]. This analysis was repeated for each species, with the three categories of PCNM variables, the environmental variables, and altitude as a single variable, resulting in five different analyses per species. We selected, for each of the three sets of PCNMs and the set of environmental variables, the variable generating the lowest possible Bayesian information criterion (BIC) value (in the fifth model, altitude was only one candidate variable). BIC values were obtained using the base R software function “BIC” [35]. The selected variable was added to the model following a forward procedure, provided that i) the resulting BIC value of the model was lower after its addition, ii) the model resulting from the added variable was significant (i.e., *P* < 0.05) following a chi-square test on a deviance table comparing the model with and without the added variable, and iii) inclusion of the variable did not preclude the algorithm from converging and did not generate fitted probabilities numerically indistinguishable from zero or one. The chi-square test on the deviance tables was performed using the function “anova” of the R base software [35]. More variables were subsequently added following the same procedure until lower BIC values could not be obtained or adding a variable did not result in significance in the chi-square test. The resulting overall fit of each of the five models for each species was then computed as the adjusted deviance value (adj-D^2^), obtained with the function “Dsquared” of the R package “modEvA” [44]. Finally, for each species, the unique adj-D^2^ value for each of the five models (i.e., unshared with that of the other four models) was obtained through deviance partitioning, analogous to the use of variance partitioning in multivariate scenarios [10, 45]. This final step allowed an estimation of the unshared explanatory ability of spatial structures (three different models for large, medium, and small spatial scales), environmental variables (one model), and altitude (one model) for the presence/absence distribution of each nematode species.

### Comparative analyses across species

**Fig 3:**
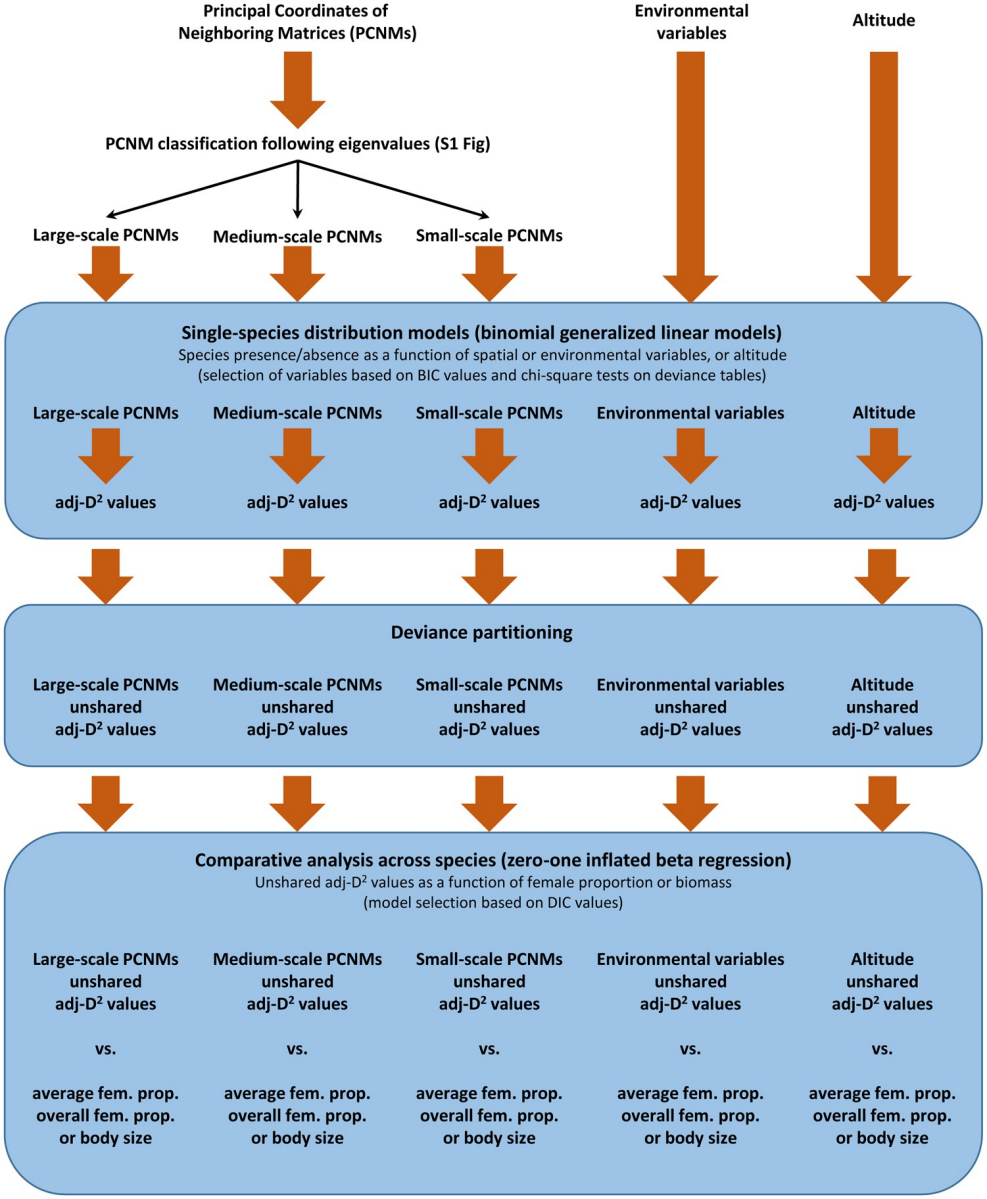
Schematic representation of the statistical analysis evaluating the ability of large-, medium-, and small-scale spatial autocorrelation and environmental variables to explain the distribution of nematode species as a function of either female proportion or body size.

The unshared adj-D^2^ values obtained by single-species modeling with the different variable subsets were compared across species within each variable subset to determine whether they were related to the proportion of nematode females and body size. More specifically, the adj-D2 values of all species resulting from each of the variable subsets were regressed on the average proportion of females, the overall proportion of females, and body size (Fig 3). Large-scale and small-scale PCNMs operating throughout the region were used to assess the potential influence of dispersal limitation and dispersal surplus, respectively, on nematode distributions. Medium-scale PCNMs were tested as an intermediate spatial autocorrelation scenario between large-scale and small-scale PCNMs. Environmental variables and altitude were not expected to influence the presence/absence of nematode species following their high tolerance to environmental changes across altitude [16]; therefore, no effect of the proportion of females or body size on the unshared adj-D^2^ values was expected for these two variable subsets. Models based on large-scale spatial variables have the potential to reveal dispersal limitation: i) if a low female proportion limits dispersal, higher unshared adj-D^2^ values would be expected for nematode species with a lower proportion of females; ii) if large body size limits dispersal, higher adj-D^2^ values would be expected for the largest nematode species.

Because adj-D^2^ values are expressed as a percentage, Gaussian linear regression models were not suitable to analyze the question at hand. Instead, percent values were divided by 100 to obtain a value between 0 and 1 that could then be used in a beta regression analysis. Since a standard beta regression precludes exact values of 0 and/or 1, both of which were possible, it was replaced by a zero-one inflated beta regression (zoib) as described in Liu and Kong [46] and performed using the R package “zoib” [47]. In the zoib regression, both a simple linear and a quadratic influence of the independent variable were considered. The quadratic version was tested for the possibility of a more flexible response. Deviance information criterion (DIC) values were used to assess the likelihood of the model [48, 49], with the best candidate model (null, simple or quadratic) defined as the one with the lowest DIC. Based on the simulations executed in this study, the model with the lowest DIC (either simple or quadratic) was selected over the null model with no variables provided that the candidate model resulted in a DIC value at least 4 units lower. A simple Spearman correlation test was used as a complementary procedure to support the validity of the models selected with the zoib regression.

Finally, generalized additive models (GAMs) were constructed to understand better the influence of female proportion and body size on geographically clustered distributions. Moran’s *I* spatial autocorrelation index of each species served as the dependent variable. The average female proportion, the overall female proportion, and the typical body size of nematode species as the independent variables, were tested individually. GAMs were chosen because of their flexibility [50] and were performed using the R package “gam” [51]; the analysis also provided information about the significance of parametric and non-parametric effects. The resulting BIC value of each model was compared with that of the null model with no variables. A Gaussian response of the Moran’s *I* values was assumed, and an identity link function and a smoothing spline of 4 degrees of freedom were used.

We explored the potential influence of nematode’s species abundance (as a surrogate of density) on the ability of the different subsets of variables to account for species distributions, and the influence of abundance on Moran’s *I* values, by a series of Spearman correlation tests. Abundance was considered as average abundance per species (either excluding zeros or including all abundance values), and as maximum abundance per species.

### Females and body size across elevation

The proportion of females and the average body size of all nematode species combined in each of the 75 lakes were also investigated concerning altitude. Since dispersal limitations were expected to be stronger at the highest elevations, and if a low proportion of females limits dispersal, the proportion of females should be highest at the highest lakes. Likewise, if large body sizes limit dispersal, a smaller average body size should be expected at the highest lakes. Again, a zoib regression was used to test the influence of altitude on the proportion of females, for the same reason as discussed above. The proportion of females was considered: i) as the average proportion among all species present in a particular lake, with one value per species used to compute the average; and ii) as the overall proportion present in that lake, regardless of the species, which is equivalent to weighing up the first analysis by the relative species abundance at each lake. When the female proportion was computed as the average between species at a given lake, the requirement that at least six adults are needed applied independently for each species at that lake.

Even though body size values are not constrained to values between 0 and 1, a zoib regression was used because it conferred consistency in the statistical analyses of female proportion and body size and thus allowed comparisons of the results. As in the analysis with the proportion of females, average body size was also considered: i) as the average between all species present in a particular lake, with one value per species used to compute the average; and ii) as the overall average body size, regardless of the species, which is equivalent to weighing up the first analysis by species relative abundance at each lake. In order to adapt body size values to a zoib regression, all values (one per lake) were relativized to the largest value observed, expressed as a value of 1.

As in the comparative analysis across species, in the analysis of both females and body size across altitude the model with the lowest DIC (either simple or quadratic) was selected over the null model with no variables, provided that the candidate model resulted in a DIC value at least 4 units lower. Simple Spearman correlation tests were likewise performed to support the validity of the models selected with the zoib regression.

## Results

### Females, body size, and spatial autocorrelation in nematode species

Among the 4131 detected individuals, there were 3018 adults, with the overwhelming majority in most species being female (Table 1). In fact, of the 31 determined species, 19 included at least 80% females, and in 11 species > 90% were female. Typical body size values ranged from 0.8 μg in *Achromadora terricola*, *Aphanolaimus aquaticus*, and *Monhystera* cf. *paludicola*, to 77.4 μg in *Anatonchus dolichurus* (Table 1). As with female proportion, the distribution of body size values was uneven, with only four nematodes having a body size > 20 μg (range: 22–77 μg) but 23 nematode species having a body size < 10 μg. Twenty of the detected nematode species were present in at least four lakes and included at least six adults; these 20 nematode species were therefore subjected to further statistical analysis. According to the Moran’s *I* spatial autocorrelation index, four nematode species had a significant spatial autocorrelation (Table 1). Distribution maps of the 20 nematode species used in statistical analysis, along with their Moran’s *I*, are shown in S2 Appendix.

**Table 1:** Species found in the lake survey (n = 75).

Species	freq.	ab.	ad.	AFP	OFP	B	*I*
*Achromadora terricola* (T)	2	4	4	1.000	1.000	0.782	-
*Anatonchus dolichurus* (S)	12	112	36	1.000	1.000	77.429	0.045
*Aphanolaimus aquaticus* (S, L)	2	3	3	1.000	1.000	0.816	-
*Aporcelaimellus obtusicaudatus* (T)	24	53	42	0.902	0.929	9.986	0.000
*Clarkus papillatus* (T, S)	2	2	2	1.000	1.000	1.390	-
*Coomansus zschokkei* (T)	9	14	8	0.583	0.625	13.062	0.033
*Crocodorylaimus flavomaculatus* (S, L)	9	36	32	0.669	0.594	1.582	-0.002
*Dorylaimus stagnalis* (L)	42	1509	1088	0.626	0.659	21.875	0.012
*Dorylaimus* sp.	8	130	111	0.651	0.604	42.497	-0.045
*Epidorylaimus consobrinus* (T, S)	44	382	332	0.999	0.997	1.541	0.028
*Ethmolaimus* cf. *pratensis* (L)	4	7	6	0.167	0.333	1.157	**0.144**
*Eudorylaimus similis* (S)	7	11	9	1.000	1.000	6.714	0.020
*Eutobrilus grandipapillatus* (S, L)	31	467	303	0.838	0.818	4.463	**0.088**
*Ironus longicaudatus* (S, L)	17	205	159	0.993	0.987	0.997	-0.016
*Ironus tenuicaudatus* (T, S, L)	16	269	219	0.566	0.548	10.463	**0.138**
*Ischiodorylaimus* cf. *cognatus* (L)	1	4	4	0.000	0.000	39.555	-
*Mesodorylaimus* cf. *conurus* (L)	4	7	7	0.792	0.714	2.844	0.056
*Monhystera* cf. *paludicola* (L)	2	5	4	0.667	0.500	0.815	-
*Mononchus truncatus* (S, L)	19	110	91	1.000	1.000	4.944	0.028
*Paractinolaimus macrolaimus* (S, L)	16	170	138	0.766	0.536	10.819	**0.111**
*Plectus aquatilis* (L)	8	35	23	1.000	1.000	1.303	-0.030
*Plectus cirratus* (L)	4	5	1	1.000	1.000	2.449	-
*Prionchulus muscorum* (T, S, L)	2	2	1	1.000	1.000	6.537	-
*Prionchulus* cf. *punctatus* (T, S, L)	1	1	1	1.000	1.000	6.859	-
*Prodorylaimus filiarum* (T,S)	1	12	9	0.222	0.222	2.363	-
*Prodorylaimus* cf. *rotundiceps* (T, S, L)	5	17	9	1.000	1.000	3.426	-0.033
*Semitobrilus pellucidus* (L)	15	46	35	0.929	0.943	2.649	0.055
*Tobrilus gracilis* (S, L)	8	45	36	0.804	0.833	6.315	0.064
*Tobrilus* sp.	1	3	3	1.000	1.000	3.375	-
*Tripyla filicaudata* (T, S)	1	15	11	0.909	0.909	1.528	-
*Tripyla glomerans* (T, S, L)	35	302	234	0.669	0.624	17.787	-0.032
Mermithidae	31	137	56	0.059	0.018	5.556	-
Indeterminate	6	11	1	1.000	1.000	0.125	-

Frequency of occurrence (freq.), total abundance (ab.), number of adults (ad.), average female proportion (AFP), overall female proportion (OFP), and average body size (B, μg) are shown, except for Mermithidae and indeterminate individuals for which body size was not estimated. For species present in at least four lakes and represented by at least six adults (n = 20), Moran’s index (*I*) is shown (boldface for *P* < 0.05). Distribution maps of those species are provided in S2 Appendix. Terrestrial (T), semiaquatic (S), or limnic (L) species are indicated [40–42].

### Single-species models

The binomial GLM results (Table 2) indicated that the presence/absence of nematode species was, in all cases, significantly affected by spatial variables at one or more of the examined spatial scales (large, medium, small). The frequency of the association of the different spatial scales with nematode distributions was more or less even across species: 12 species associated with one or more large-scale spatial variables, 15 with medium-scale variables, and 9 with small-scale variables. The distribution of many nematode species was associated with more than one spatial scale, and three nematode species (*Prodorylaimus* cf. *rotundiceps*, *Semitobrilus pellucidus*, and *Tripyla glomerans*) associated with all three spatial scales. While most nematode species were affected to some extent by one or more environmental variables, usually water chemistry or productivity descriptors, only one (*Dorylaimus stagnalis*) correlated significantly (inversely) with altitude. In a few cases, the addition of subsequent variables into the different models generated fitted probabilities numerically indistinguishable from 0 or 1 in one or more lakes (which could be indicative of overfitting), and on one occasion the subsequent addition of a variable resulted in the algorithm’s failed convergence. However, these issues were readily solved in all cases by replacing the variable in question by the next one on the list of BIC values, provided that chi-square significance (*P* < 0.05) on a deviance table was also achieved (see footnotes in Table 2 for details on these few cases).

**Table 2:** Binomial generalized linear models (GLMs) incorporating large-, medium-, and small-scale principal coordinates of neighboring matrices (PCNMs), environmental variables, and altitude (as a separate variable) to predict the presence/absence of nematode species.

Species	Large PCNMs	Medium	Small	Env.	Altitude
		PCNMs	PCNMs	variables	
*Anatonchus dolichurus* ^1^	PCNM-1		PCNM-35	Calcium (−)	
	PCNM-6			Temperature (+)	
				TP (−)	
				Potassium (−)	
*Aporcelaimellus obtusicaudatus*		PCNM-25			
		PCNM-13			
*Coomansus zschokkei*	PCNM-1		PCNM-39	Sodium (−)	
			PCNM-45	Chl-*a* (+)	
*Crocodorylaimus flavomaculatus*		PCNM-27	PCNM-38	Chloride (+)	
*Dorylaimus stagnalis*	PCNM-6			ANC (+)	Altitude (−)
				Nitrate (−)	
				Lake Area (+)	
*Dorylaimus* spec ^2^		PCNM-28	PCNM-36	Chl-*a* (+)	
		PCNM-25		Bacteria (−)	
				pH (+)	
*Epidorylaimus consobrinus*	PCNM-2			Magnesium (−)	
*Ethmolaimus* cf. *pratensis* ^3^	PCNM-6	PCNM-9			
	PCNM-2				
*Eudorylaimus similis*		PCNM-19		Bacteria (−)	
*Eutobrilus grandipapillatus*	PCNM-3			Chl-*a* (+)	
	PCNM-2			Lake Depth (+)	
*Ironus longicaudatus*		PCNM-11			
*Ironus tenuicaudatus*	PCNM-1	PCNM-24		Salmonidae (+)	
*Mesodorylaimus* cf. *conurus* ^4^	PCNM-5	PCNM-12			
		PCNM-11			
*Mononchus truncatus*		PCNM-12	PCNM-45	Silicon (−)	
*Paractinolaimus macrolaimus*	PCNM-6	PCNM-26		Calcium (−)	
	PCNM-1			TN (−)	
*Plectus aquatilis*		PCNM-17	PCNM-37	Potassium (−)	
		PCNM-25		Gravel (−)	
		PCNM-21		Stones (+)	
*Prodorylaimus* cf. *rotundiceps* ^5^	PCNM-5	PCNM-30	PCNM-39	K (−)	
				LOI (+)	
*Semitobrilus pellucidus*	PCNM-2	PCNM-11	PCNM-38	Conductivity (−)	
				Potassium (−)	
*Tobrilus gracilis*		PCNM-10		Lake Area (+)	
		PCNM-24			
*Tripyla glomerans*	PCNM-6	PCNM-17	PCNM-42	Chl-*a* (+)	
		PCNM-19		DOC (−)	
				TP (+)	
				*Phoxinus* (−)	

Variables in each of the models are shown according to their order of selection following the Bayesian information criterion (BIC), provided they were also significant (*P* < 0.05) in a chi-squared test on a deviance table. Signs in parentheses after the environmental variables and altitude indicate a positive or negative response for each species. Only nematodes present in at least four lakes and represented by at least six adults (n = 20) were considered (see text).

^1^ In the environmental model, potassium (−) replaced macrophytes (+) as the last environmental variable selected; macrophytes was the preferred variable following BIC values but it generated three fitted probabilities (in three lakes) numerically indistinguishable from 0.

^2^ In the environmental model, BIC values suggested the selection of an additional variable, magnesium (−), but it was discarded because it generated fitted probabilities numerically indistinguishable from 0 (n = 3).

^3^ In the environmental model, BIC values suggested the selection of DOC (−) and TP (−); however, both were discarded as they generated fitted probabilities numerically indistinguishable from 0 (n = 1, in both cases).

^4^ In the medium-scale PCNM model, BIC values suggested the additional selection of PCNM-16 or PCNM-21; however, both were discarded as they generated fitted probabilities numerically indistinguishable from 0 or 1 (n = 70 in the case of PCNM-16, in which, additionally, the algorithm did not converge, and n = 2 in the case of PCNM-21).

^5^ In the environmental model, BIC values suggested the selection of an additional variable, Gravel (+); however, the inclusion of this variable was discarded as it generated one fitted probability numerically indistinguishable from 0.

Calculations of the overall deviance accounted for by the full binomial GLMs, species by species (i.e., incorporating each of the variables selected in each of the five different binomial GLMs for each of the 20 species) yielded values ranging from 7.4% (*Aporcelaimellus obtusicaudatus*) to 75.4% (*Coomansus zschokkei*) (Table 3). Deviance partitioning likewise revealed large differences in the influence of spatial and environmental variables among species (Table 3). The only species that correlated significantly with altitude (*Dorylaimus stagnalis*) did not respond to the unshared fraction of altitude when this variable was merged with the others in the full binomial GLM. In fact, there was a negative value for the unshared deviance accounted for by altitude, which suggested that altitude is affected by other variables in explaining the distribution of this species. In view of the results obtained with altitude, both in the binomial GLMs (Table 2) and in deviance partitioning (Table 3), this variable was discarded from further statistical analysis.

**Table 3:** Partitioning of the adjusted-D^2^ (adj-D^2^) values accounted for by binomial GLMs predicting nematode presence/absence with large-, medium-, and small-scale PCNMs, environmental variables, and altitude.

Species	Total	Shared	Large PCNMs (unshared)	Medium PCNMs (unshared)	Small PCNMs (unshared)	Environ. variables (unshared)	Altitude (unshared)
*Anatonchus dolichurus*	56.74	11.02	2.03	0.00	3.46	40.23	0.00
*Aporcelaimellus obtusicaudatus*	7.40	0.00	0.00	7.40	0.00	0.00	0.00
*Coomansus zschokkei*	75.41	–20.53	23.63	0.00	49.25	23.05	0.00
*Crocodorylaimus flavomaculatus*	36.42	1.39	0.00	14.83	13.70	6.51	0.00
*Dorylaimus stagnalis*	14.56	2.32	4.63	0.00	0.00	8.57	-0.97
*Dorylaimus* sp.	47.53	6.96	0.00	15.46	0.24	24.86	0.00
*Epidorylaimus consobrinus*	8.87	–1.60	4.90	0.00	0.00	5.57	0.00
*Ethmolaimus* cf. *pratensis*	65.07	17.59	45.00	2.48	0.00	0.00	0.00
*Eudorylaimus similis*	28.45	8.29	0.00	5.85	0.00	14.30	0.00
*Eutobrilus grandipapillatus*	13.47	5.79	5.20	0.00	0.00	2.48	0.00
*Ironus longicaudatus*	7.99	0.00	0.00	7.99	0.00	0.00	0.00
*Ironus tenuicaudatus*	28.37	0.97	12.37	9.23	0.00	5.79	0.00
*Mesodorylaimus* cf. *conurus*	61.81	6.06	9.98	45.77	0.00	0.00	0.00
*Mononchus truncatus*	15.88	–0.40	0.00	6.02	4.95	5.31	0.00
*Paractinolaimus macrolaimus*	45.83	3.99	6.20	8.36	0.00	27.29	0.00
*Plectus aquatilis*	73.46	5.02	0.00	32.97	4.41	31.07	0.00
*Prodorylaimus* cf. *rotundiceps*	59.23	13.02	3.19	9.35	13.55	20.13	0.00
*Semitobrilus pellucidus*	31.44	7.35	-0.05	8.70	7.44	8.00	0.00
*Tobrilus gracilis*	25.63	1.95	0.00	17.61	0.00	6.08	0.00
*Tripyla glomerans*	34.34	–3.65	5.54	9.04	3.95	19.46	0.00

Total adj-D^2^ values result from merging the variables selected in each of the five independent binomial GLMs for each species in a full binomial GLM. The partitioning of adj-D^2^ values into shared and unshared fractions is shown. Only nematodes present in at least four lakes and represented by at least six adults (n = 20) were considered.

### Comparative analyses across species

The influence of female proportion and body size on the unshared deviance accounted for by the different models was compared across species in a zoib regression, excluding altitude for the reasons discussed above. Therefore, we explored the influence of the average proportion of females, the overall proportion of females, and the typical body size on the unshared deviance accounted for by large-scale, medium-scale, and small-scale spatial variables and environmental variables. Only three models fulfilled the requirement of having a DIC value at least 4 units lower than that of the null model: 1) a simple model for the association between large-scale variables with the average proportion of females; 2) a quadratic model version in the case of the overall proportion of females; and 3) a simple model for the association between environmental variables and body size (Table 4).

**Table 4:** Deviance Information Criterion (DIC) values of zero-one inflated beta regression models across species (adjusted deviance of each species accounted for by different subsets of variables regressed on female proportion or body size).

	Average females	Overall females	Body size
**Large-scale PCNMs**			
*Null*	40004.85	40004.81	40004.84
*Simple*	39987.38 (**–17.47**)	39994.86 (–9.95)	40005.14 (+0.30)
*Quadratic*	39989.55 (–15.30)	39992.70 (**–12.11**)	40007.52 (+2.68)
*Spearman’s rho*	–0.589 (*P* = 0.006)**	–0.554 (*P* = 0.011)*	0.137 (*P* = 0.564)
**Medium-scale PCNMs**			
*Null*	39992.64	39992.63	39992.66
*Simple*	39994.17 (+1.53)	39994.53 (+1.90)	39992.54 (–0.12)
*Quadratic*	39993.95 (+1.31)	39989.91 (–2.72)	39994.50 (+1.84)
*Spearman’s rho*	–0.014 (*P* = 0.954)	0.008 (*P* = 0.972)	–0.195 (*P* = 0.409)
**Small-scale PCNMs**			
*Null*	40009.62	40009.61	40009.56
*Simple*	40011.34 (+1.72)	40011.45 (+1.84)	40008.10 (–1.46)
*Quadratic*	40009.05 (–0.57)	40013.06 (+3.45)	40008.65 (–0.91)
*Spearman’s rho*	0.154 (*P* = 0.516)	0.182 (*P* = 0.441)	0.038 (*P* = 0.874)
**Environmental variables**			
*Null*	39992.14	39992.21	39992.12
*Simple*	39993.60 (+1.46)	39994.08 (+1.87)	39984.71 (**–7.41**)
*Quadratic*	39993.27 (+1.13)	39992.49 (+0.28)	39986.58 (–5.54)
*Spearman’s rho*	0.132 (*P* = 0.578)	0.111 (*P* = 0.641)	0.526 (*P* = 0.017)*

Female proportion is considered either as the average proportion of females per lake (i.e., mean of proportions) or as the overall proportion of females in all lakes combined (i.e., proportion of all individuals pooled). Simple and quadratic models are compared to a null model with no variables in each case, and the one with the lowest DIC value selected, provided that this value is at least 4 units lower than in the null model (difference indicated in parentheses, highlighted in boldface for the models selected). Spearman’s *rho* and its associated *P*-value are indicated as a complementary analysis in each case (*, *P* < 0.05; **, *P* < 0.01).

**Fig 4:**
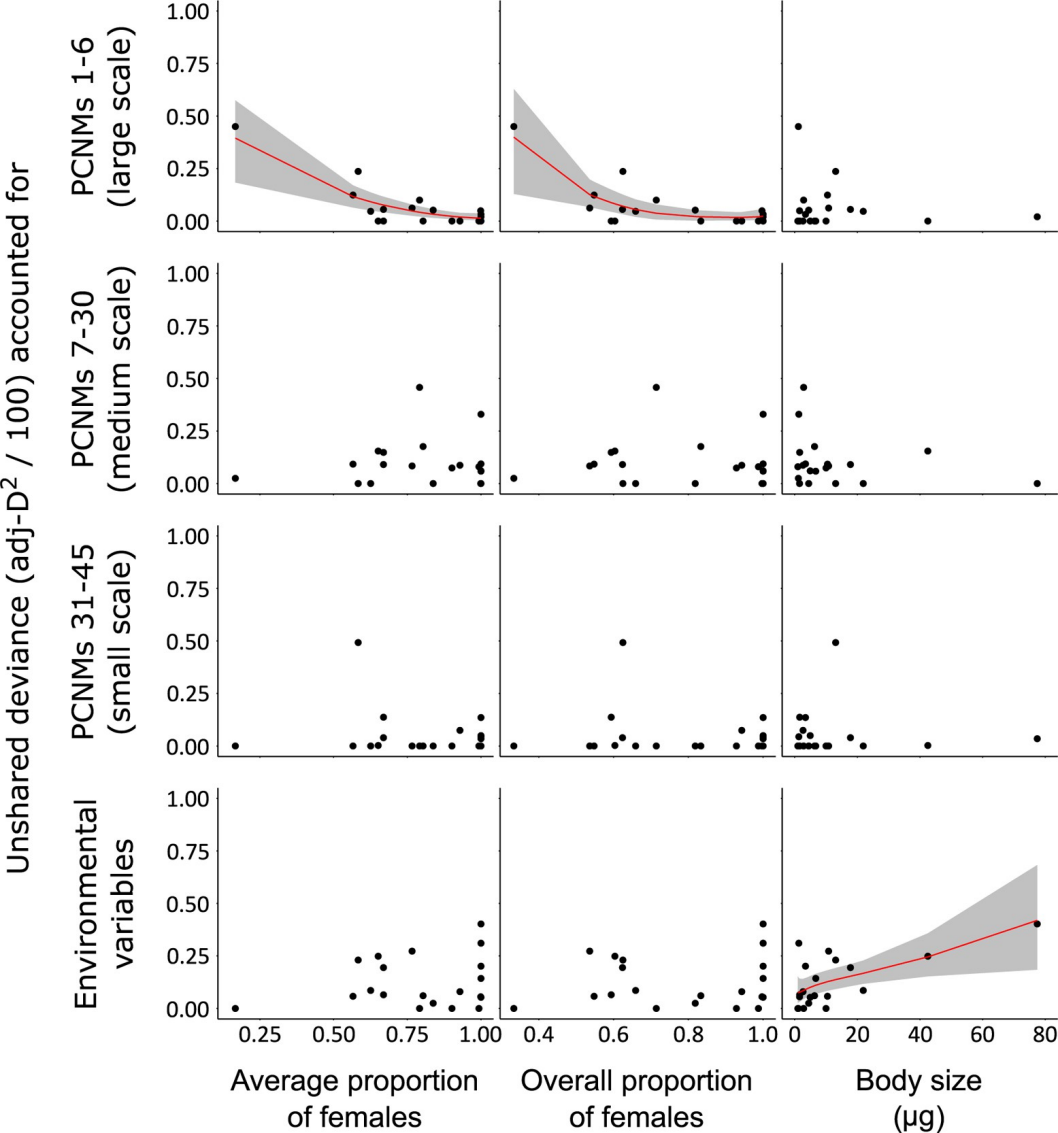
Comparative analysis across species with zero-one inflated beta regression of the unshared adj-D^2^ values of the average female proportion, overall female proportion, and average body size. The unshared adj-D^2^ values refer to deviance partitioning after the execution of four different binomial generalized linear models for each species, comparing large-, medium-, and small-scale spatial autocorrelation variables and environmental variables (see Fig 3).

The results showed that a decreasing proportion of females (as an average or overall proportion) was related to an increase in the deviance accounted for by large-scale spatial variables. Female proportion values did not distribute equally within the range of observed values, and the observed tendency appeared strongly conditioned by one extreme data point (Fig 4). However, the pattern was supported by the Spearman correlation tests: *rho* = –0.589 (*P* = 0.006) and *rho* = –0.554 (*P* = 0.011), for the average and overall proportion of females, respectively. Moreover, zoib regression was still selected over the null model with no variables after removing the extreme data point, and the association between female proportion and the deviance accounted for by large-scale spatial variables was still significant after the removal: *rho* = –0.509 (*P* = 0.026) and *rho* = –0.471 (*P* = 0.042), when using average and overall proportion of females, respectively.

In contrast to females, body size was not related to the ability of large-scale autocorrelation structures to account for nematode distributions but rather to the influence of environmental variables. As in the case of females, body size values did not distribute equally within the range of observed values, but were instead strongly governed by the influence of two nematode species (*Anatonchus dolichurus* and *Dorylaimus* sp.) with large body sizes. For both, the influence of environmental variables was evident in the binomial GLMs and deviance partitioning. In spite of the strong influence of two species, the result was supported by a Spearman correlation test (*rho* = 0.526, *P* = 0.017). After removing the most extreme data point (*A*. *dolichurus*), the result was marginally significant (*rho* = 0.447, *P* = 0.055).

**Fig 5:**
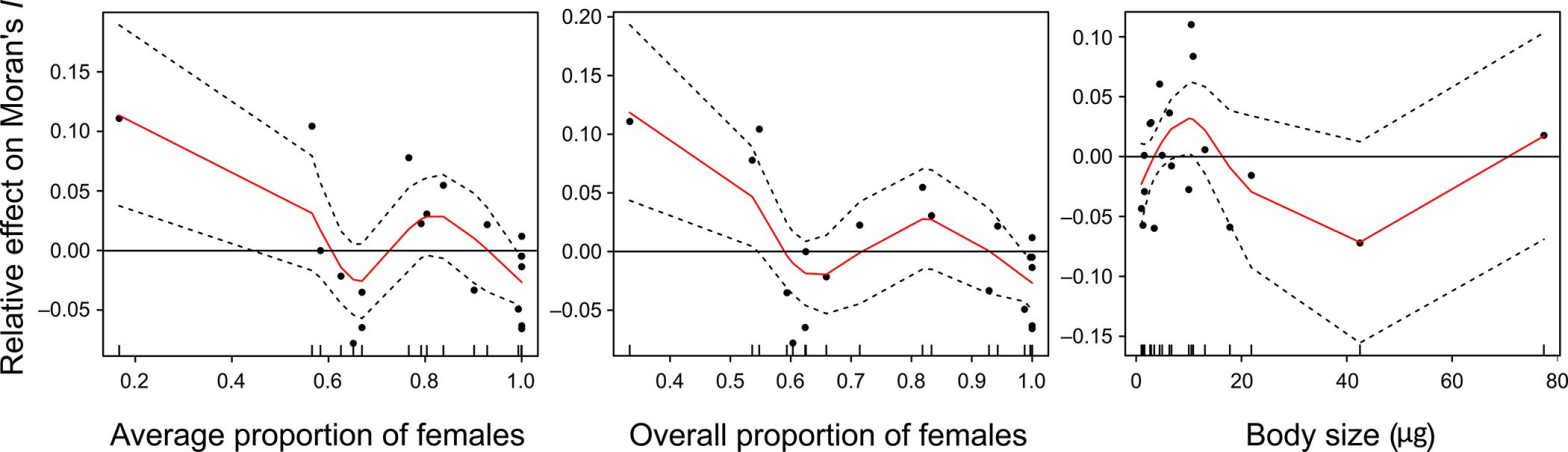
Generalized additive models with species’ Moran’s *I* as the dependent variable and either average female proportion, overall female proportion, or average body size as the independent variable. A smoothing spline of 4 degrees of freedom was used, and a Gaussian link for the response variable was assumed. The effect of female proportion (independent of how it was measured) was significant (*P* < 0.05) concerning parametric and non-parametric effects, and the inclusion of the variable was also favored based on the Bayesian information criterion (BIC), whereas this was not the case for average body size. Dotted lines indicate standard errors of the prediction. The long horizontal solid line represents a zero effect. Short vertical upward ticks on the x-axis indicate different values of the independent variables.

GAMs performed on the Moran’s *I* spatial autocorrelation index showed that a lower average or overall proportion of females was significantly related to an increase in the spatial autocorrelation of nematodes, whereas this was not the case for body size (Table 5, Fig 5). The significance of the average (overall) proportion of females concerning parametric and non-parametric effects was *P* = 0.012 (*P* = 0.013) and *P* = 0.013 (*P* = 0.013), respectively, and BIC values were lower for average and overall proportion of females (–63.7 and –63.6, respectively) than that of a null model with no variables (–54.0). Parametric effects became not significant after removing the most extreme data point (*P* = 0.202 and *P* = 0.104, for average and overall proportion of females, respectively), yet non-parametric effects were still significant (*P* = 0.003 and *P* = 0.004, respectively), BIC values still lower (–65.6 and –65.0, respectively) than in the null model (–54.9), and the adjusted deviance accounted for by either the average or the overall proportion of females was still higher than 50%, and still significant (i.e., *P* < 0.001) following a chi-square test on a deviance table. For body size, parametric and non-parametric effects were not significant (*P* = 0.609 and *P* = 0.273, respectively), BIC values were higher than in the null model (–50.3 vs –54.0), and the adjusted deviance accounted for was lower than 20% and not significant (i.e., *P* > 0.05). These results for body size remained unaltered after the removal of the most extreme body size value.

**Table 5:** Generalized additive models (GAMs) across species, with the Moran’s *I* as the response variable, and either female proportion or body size as the independent variable.

	Females (average)	Females (overall)	Body size
BIC (null model)	–53.99	–53.99	–53.99
BIC (with the variable)	–63.71 (–9.71)	–63.59 (–9.59)	-50.31 (+3.69)
Parametric effects	*P* = 0.012 *	*P* = 0. 013 *	*P* = 0.609
Non-parametric effects	*P* = 0.013 *	*P* = 0. 013 *	*P* = 0.273
adj-D^2^	56.1%	55.9%	14.3%
Chi-square test	*P* < 0.001 ***	*P* < 0.001 ***	*P* = 0.335

Female proportion is considered either as the average proportion of females per lake (i.e., mean of proportions) or as the overall proportion of females in all lakes combined (i.e., proportion of all individuals pooled). In each case, Bayesian Information Criterion (BIC) values are shown for the model obtained and the null model with no variables. The difference between the two is indicated in parentheses. Significance (*P*-value) obtained for parametric and non-parametric effects is indicated. Adjusted percentage of deviance accounted for by the variable at hand (adj-D2) is also indicated, with the associated Chi-square test on the deviance table. Asterisks denote the level of significance:

*, *P* < 0.05;

***, *P* < 0.001.

We found no significant correlation (Spearman’s *rho*) between the abundance of nematode species and the power of the different subsets of variables considered to explain their distribution, and there was no correlation between abundance and Moran’s *I* either (S1 Table).

### Females and body size across elevation

**Fig 6:**
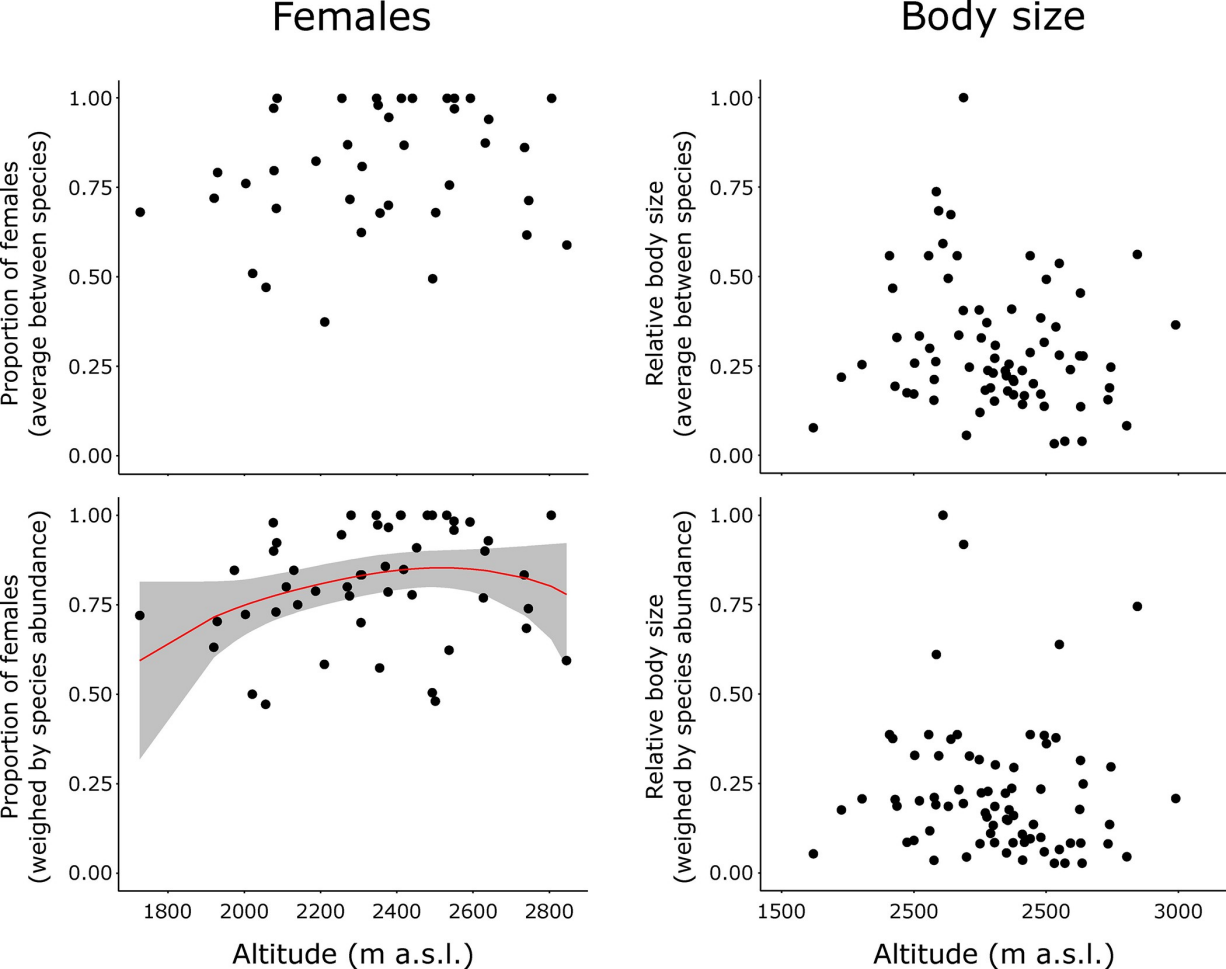
Proportion of females (left) and body size (right) at each lake, across altitude, following a zero-one inflated beta regression analysis. Female proportion was estimated either as average between species at each lake (only species with at least six adults in a given lake were considered), or as values weighed by species abundance at each lake (only lakes with at least 6 adults, independently of the species, were considered). Female proportion tends to increase across elevation when estimated as values weighed by species abundance at each lake, but the pattern is not well supported by a Spearman correlation test (*rho* = 0.236; *P* = 0.095). No pattern was observed for body size. Data available in S2 Table.

Assuming that the highest elevations impose the strongest limitations on dispersal, we evaluated the influence of lake altitude on the proportion of females and the average nematode body size. In both cases, we used a zoib regression to facilitate comparisons. As expected, the distribution of the data on female proportion and body size within the range of possible values was more balanced since an analysis focused on lakes instead of on species implies an analysis of communities of coexisting nematodes, such that the influence of extreme values (e.g., nematode species with very low proportions of females or very large body sizes) is diluted. In the case of female proportion, some data were discarded as there was no sufficient amount of adults per lake, reducing sample size to 39 and 51 lakes, for the average between species, and values weighed by species abundance, respectively. The altitudinal gradient covered with this sample size reduction was only slightly compromised (i.e., from 1620–2990 to 1726–2845 m a.s.l., in both cases). There was a slight tendency of an increase across altitude following the zoib regression but only when computed as an overall value, weighed by species abundance, at each lake (Table 6, Fig 6, S2 Table). The pattern, however, was not well supported by a Spearman correlation test (n = 51, *rho* = 0.236, *P* = 0.095). When computed as average values between species at each lake, Spearman correlation was not significant (n = 39, *rho* = 0.212, *P* = 0.196). There was no altitudinal pattern relating to body size following zoib regression (Fig 6, S2 Table). This was confirmed by Spearman correlation tests both for average values between species (n = 75, *rho* = –0.183, *P* = 0.117) and values weighed by species abundance (n = 75, *rho* = –0.190, *P* = 0.102).

**Table 6:** Deviance Information Criterion (DIC) values of zero-one inflated beta regression models across lakes (i.e., proportion of females or body size against altitude).

	Average between species	Weighed by species abundance
**Female proportion**		
*Number of lakes*	39	51
*Altitudinal range*	1726–2845	1726–2845
*Null*	78014.62	101994.56
*Simple*	78014.68 (+0.06)	101993.20 (–1.36)
*Quadratic*	78014.21 (–0.41)	101989.45 (**–5.11**)
*Spearman’s rho*	0.212 (*P* = 0.196)	0.236 (*P* = 0.095)
**Body size**		
*Number of lakes*	75	75
*Altitudinal range*	1620–2990	1620–2990
*Null*	149941.43	149929.68
*Simple*	149941.57 (+0.14)	149930.58 (+0.90)
*Quadratic*	149940.46 (–0.97)	149930.54 (+0.86)
*Spearman’s rho*	–0.183 (*P* = 0.117)	–0.190 (*P* = 0.102)

Simple and quadratic models are compared to a null model with no variables, and the one with the lowest DIC value selected, provided that this value is at least 4 units lower than in the null model (difference indicated in parentheses, highlighted in boldface for the models selected). Spearman’s *rho* and its associated *P*-value are indicated as a complementary analysis. Female proportion and body size are considered at each lake either as the average value between present species or as values weighed by species abundance. In the case of female proportion, average values between species at a given lake were estimated using only species for which at least 6 adults were found in that particular lake. Female values weighed by species abundance were estimated only for lakes with at least 6 adults. The number of lakes used and the altitudinal range covered (m a.s.l.) are also indicated.

## Discussion

Our results suggested that female proportion is more influential than body size on nematode’s long-distance passive dispersal. Thus, for nematode species inhabiting mountain lakes, successful colonization via passive dispersal may depend more on a high female proportion than on a small body size. Specifically, the clustered geographical distributions were significantly related to a low proportion of females but not to body size. This finding was supported by the significant inverse relationship between the proportion of females in each species and the ability of large-scale spatial variables to account for species distributions, an observation validated by the analysis of Moran’s *I* but not found for body size (Figs 4 and 5). This inverse relationship, while sustained by the effect of a few species with the lowest proportion of females, was supported by the Spearman correlation. On the other hand, altitude accounted for a slight increase in the proportion of females at a given lake (Fig 6), yet this was not supported by a Spearman test, possibly because of the quadratic component of this association, as highlighted by the zoib regression, and reduced sample size.

There was no evidence of a relationship between the influence of small-scale spatial variables on species metapopulations and the proportion of females within these populations or the body size of those species. Considering the generally high isolation between lakes population connectivity between sites is probably not strong enough to produce small-scale spatial patterns resulting from dispersal surplus. In general, very few studies have found robust indications for spatial structuring due to dispersal surplus [4], but among the few metacommunity studies of nematodes, such indications can be found, although contingent on the type of investigated environment. For example, the high dispersal rates along the watercourse of riverine networks led to the assumption that mass effects are common and produce spatial patterns [17, 27]. The results of the present study suggest that this is not the case in mountain lakes. Differences in local abundance across species did not support the idea that the density of individuals per site is influencing spatial patterns (S1 Table).

### The relevance of females

In freshwater environments, free-living adult nematodes are usually dominated by females and thus by (facultative) parthenogenetic species, as shown in this study (Table 1) and previous investigations [52–54]. The frequency of this reproductive mode may reflect an adaptation to isolation in many freshwater habitats. For example, in the groundwater system of a completely isolated cave, not a single male was present among the five nematode species [55]. In marine habitats, however, sexual reproduction may be obligatory for nematodes (e.g., [54, 56]). Although dispersal consists of three successive phases, emigration, transfer, and immigration [57, 58], previous studies relating dispersal ability to metacommunity structure focused on the traits affecting the transfer phase, such that the role of females and reproductive potential as important aspects of immigration (e.g., [9, 59]) have been ignored.

To the best of our knowledge, this is the first study to investigate the effect of female dominance on the dispersal ability and metacommunity structuring of a passively dispersing organismal group. By contrast, the importance of sex and the population sex-ratio in active dispersal has been examined in several studies [60, 61], especially in the context of evolutionary processes (e.g., [62–64]). Although the small-scale active dispersal of nematodes may reinforce their fundamentally passive dispersal mode, such as by guiding them to suitable transport vectors [21], the results obtained in active dispersal studies are not easily transferrable to passive dispersal, in which stochasticity plays an important role.

Future research should thus consider the dominance of females among the traits potentially relevant for dispersal, including in studies of the role of spatial structures in the geographical distribution of passively dispersing organisms. The wide variation in the reproductive modes (and resulting sex-ratios) of nematodes, both between and within species [40, 65], has been used as the starting point in evolutionary studies [65]. However, as our study has shown, it can also be exploited to investigate the role of reproductive modes in dispersal and metacommunity patterns. Moreover, the responses (and mechanisms thereof) of the sex-ratio of populations to biotic and abiotic conditions should also be taken into account, to avoid confounding effects in the analyses. For example, the sex-ratio may be related to nematode species richness [54], and to temperature and resource availability [66].

### Body size and environmental factors

The lack of a relationship between body size and spatial distribution structure in the investigated nematode species was surprising, given the evidence in previous studies that small body size increases the dispersal distance and frequency of passively dispersing organisms (e.g., [8, 15, 19]). However, there are also examples to the contrary (e.g., [67, 68]). In this study, the smallest nematodes may have been underrepresented owing to the mesh size used in sampling. However, it is unlikely that their inclusion would have changed the results, as large-scale spatial structures in nematode distribution should be more evident in larger species.

The demonstration that body size is related to the spatial distribution of a species may depend on the assumptions made about the type of transport vector relied upon by passively-dispersing organisms. If nematodes are mostly transported by wind, then the transportation rate and distance will decline with increasing particle (i.e., body) size. Yet, other vectors may also play an essential role in the overland dispersal of small organisms. For example, Jenkins *et al*. [67] suggested that in their meta-analysis, the lack of relationship between size and dispersal distance could be due to the role of zoochory (i.e., the transportation of small propagules via larger animals). Nematodes are often found on and in larger animals, e.g., waterfowl (see references in the review of Ptatscheck and Traunspurger, [21]). However, waterfowl are unlikely to be significantly present in mountain areas, particularly at the highest elevations.

Alternatively, passive transport by wind might not be restricted to “active” stages of nematodes but also include “inactive stages”, such as eggs, dauerlarvae, and anhydrobiotic stages [21]. In the latter cases, body size may not directly relate to the body size estimates of living individuals used in this study. Consequently, the ability of a nematode species to produce many eggs and resting stages able to persist under harsh conditions, such as desiccation, frost, starvation, and digestion, might be a more important species trait than body size, especially in the extreme environment of high altitudes [19]. Indeed, Baujard and Martiny [69] caught anhydrobiotic-stage nematodes in aeroplankton. However, it seems that resting stages such as dauerlarvae and anhydrobiosis are mainly restricted to largely terrestrial species and to species that colonize ephemeral aquatic habitats (e.g., ponds), as neither has yet to be detected in obligate aquatic species ([21] and literature therein). Furthermore, facultative aquatic species are likely to particularly benefit from transport overland between lakes, as they are generally more adjusted to drought and unstable conditions than obligate aquatic nematodes. Interestingly, in this study, the most clustered species, *E*. cf. *pratensis*, was also one of the obligate aquatic species detected (Table 1), thus suggesting that environmental tolerance might be relevant to dispersal. Finally, it is also possible that the advantage of a high female proportion in mountain areas minimizes any difference in body size between nematode species, a conclusion supported by the positive relationship between female proportion and elevation.

A test of whether environmental factors influence species distributions and how this effect could be related to the proportion of females or body size showed a significant positive relationship between the role of environmental filtering and nematode body size. While this pattern seems reliant mainly on the influence of two species, *Anatonchus dolichurus* and *Dorylaimus* sp., which were by far the largest and were strongly associated with environmental gradients, this result was supported by a simple Spearman correlation. If this finding had reflected a dispersal-related process, then the reverse pattern would have been expected, i.e., the better ability of species with lighter individuals and thus a higher dispersal rate to track environmental gradients, leading to a stronger association of their distribution with those gradients. However, according to our study, body size may not be particularly relevant for nematode dispersal in mountain lakes. Furthermore, spatial and environmental variables were analyzed as unique effects (i.e., after the effect of the other variables was partialled out). Therefore, the association between body size and species-environment relationships, as observed in this study, should be independent of dispersal processes. One possibility is that this pattern may reflect the higher metabolic demand of larger nematodes, such that their geographical distribution is more strongly influenced by environmental factors.

## Conclusions

Our analysis of nematodes from nutrient-poor mountain lakes, where local anthropogenic disturbance is generally very low, and nematode species hardly respond to environmental factors [16], showed the influence of dispersal-related stochastic processes on nematode species distributions and metacommunities. We hypothesized that a larger proportion of females and a smaller body size would confer dispersal advantages, but only support for the former was obtained. The absence of a relationship between body size and dispersal for nematode species in mountain lakes was in contrast to previous studies. However, in mountain areas, the passive dispersal agent might be mainly wind, which has a stronger impact on early, resistant stages in which size is not directly related to the final adult body size. Our study provides the first insights into the importance of female dominance in the dispersal of passively-dispersing organisms. Based on our findings, species traits related to the immigration phase of dispersal, and specifically female dominance, should be included in trait-based analyses to provide a deeper understanding of the processes underlying restricted species distributions and metacommunity structuring in passively dispersing organisms.

## Supporting information

S1 Fig PCNM scale classification into large, medium, and small. (PDF)

S1 Appendix Maps of the PCNMs with positive eigenvalues. (PDF)

S2 Appendix Distribution maps of the nematode species analyzed. (PDF)

S1 Table Local abundance across species in relation to deviance explained and Moran’s *I*. (PDF)

S2 Table Female proportion and body size data per lake, with geographic coordinates. (PDF)

S1 Data Nematode species data (individuals, adults, and females) per lake. (XLSX)
