# Supplementary material for: Female proportion has a stronger influence on dispersal than body size in nematodes of mountain lakes
Source: PLoS One. 2024 May 17;19(5):e0303864. doi: 10.1371/journal.pone.0303864 (PMC11101049; doi:10.1371/journal.pone.0303864)

Supporting Information for

Female proportion has a stronger influence on dispersal than body size

in nematodes of mountain lakes

G. de Mendoza, B. Gansfort, J. Catalan & W. Traunspurger

S1 Fig Principal coordinates of neighboring matrices (PCNMs) with positive eigenvalues (n = 45), with the latter used to distinguish large-scale (brown), medium-scale (green), and small-scale (purple) PCNMs (eigenvalues >25000, 1000-25000, and <1000, respectively). Eigenvalues are presented as raw data (top) and as log-transformed data (bottom).

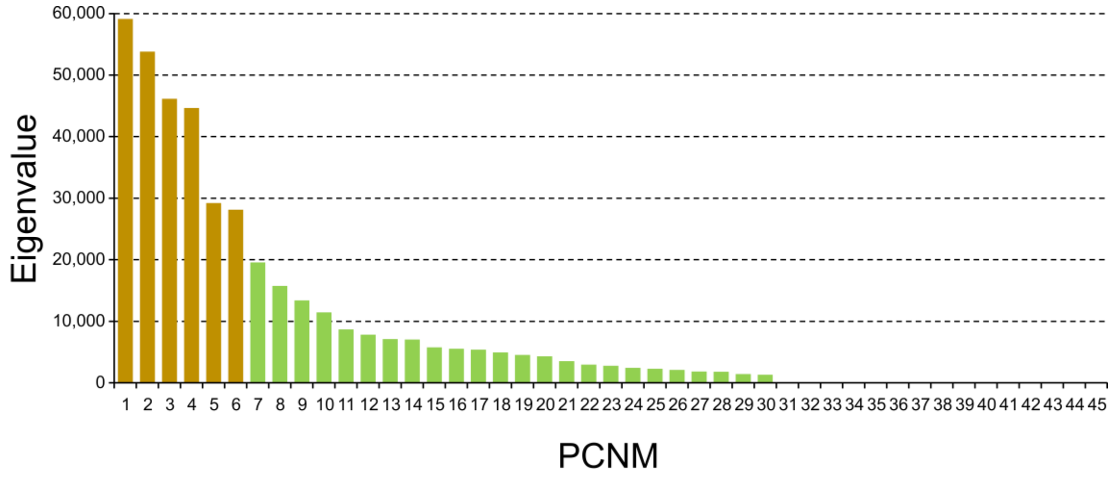

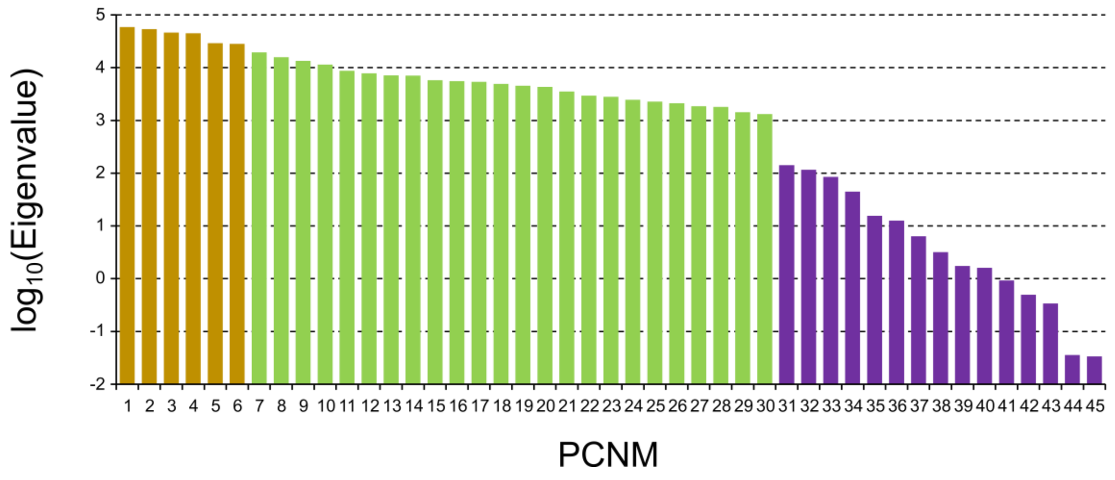

Supplement: S1 Fig — (PDF) [file pone.0303864.s001.pdf]
