# Supplementary material for: Female proportion has a stronger influence on dispersal than body size in nematodes of mountain lakes
Source: PLoS One. 2024 May 17;19(5):e0303864. doi: 10.1371/journal.pone.0303864 (PMC11101049; doi:10.1371/journal.pone.0303864)

Supporting Information for

Female proportion has a stronger influence on dispersal than body size in nematodes of mountain lakes

G. de Mendoza, B. Gansfort, J. Catalan & W. Traunspurger

S1 Appendix Maps of the 45 principal coordinates of neighboring matrices (PCNMs) with positive eigenvalues, obtained based on the geographical distances between lakes (n = 75). Maps represent the Pyrenees in Cartesian space, which was used to build the PCNMs, with a threshold distance equivalent to the maximum distance in a minimum spanning tree, keeping all lakes connected. Maps are presented for large-scale PCNMs (PCNM-1 to PCNM-6), medium-scale PCNMs (PCNM-7 to PCNM-30), and small-scale PCNMs (PCNM-31 to PCNM-45).

S1 Appendix Maps of PCNMs: large-scale PCNMs, from PCNM-1 to PCNM-6.

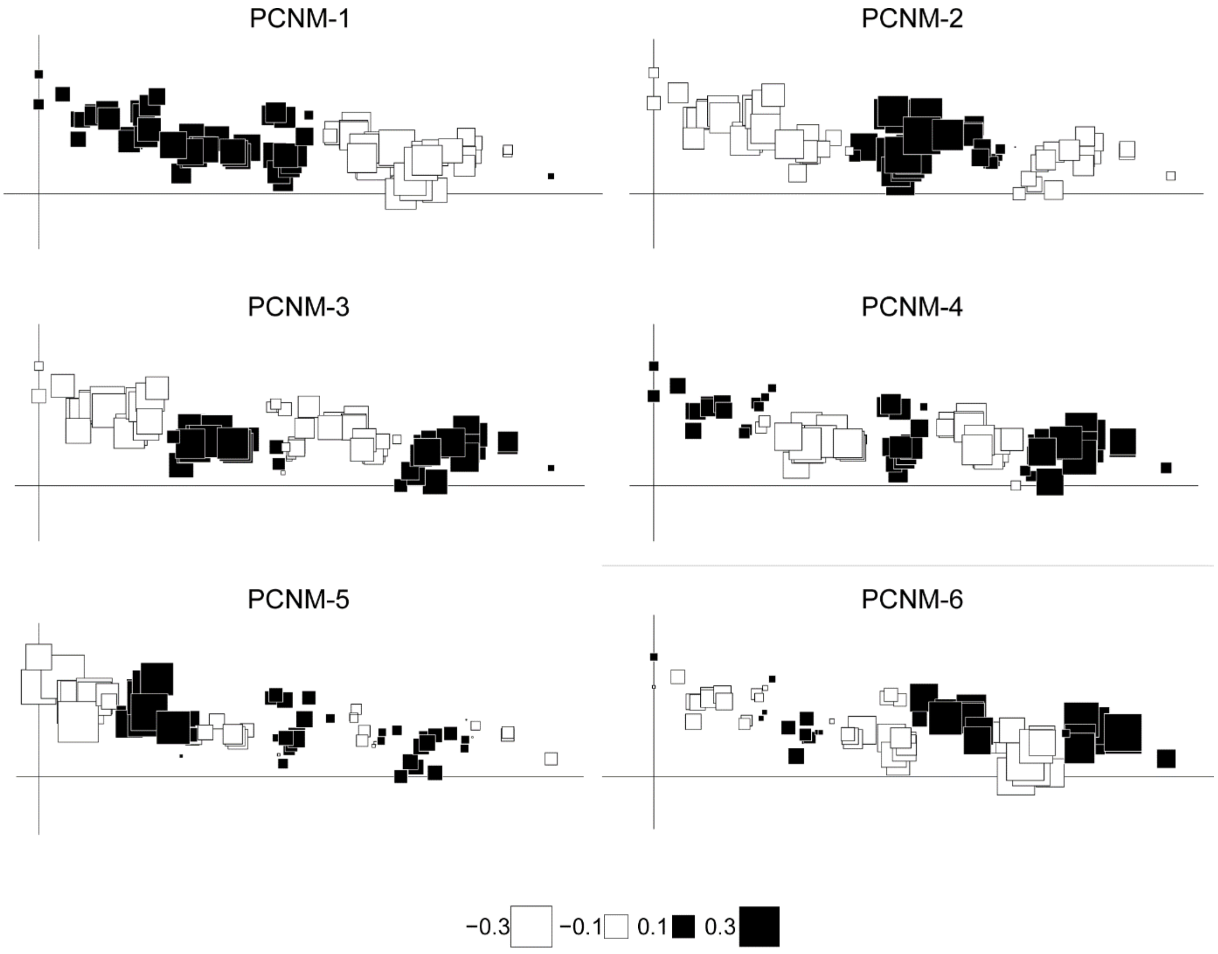

S1 Appendix (continued) Maps of PCNMs: medium-scale PCNMs (I), from PCNM-7 to PCNM-18.

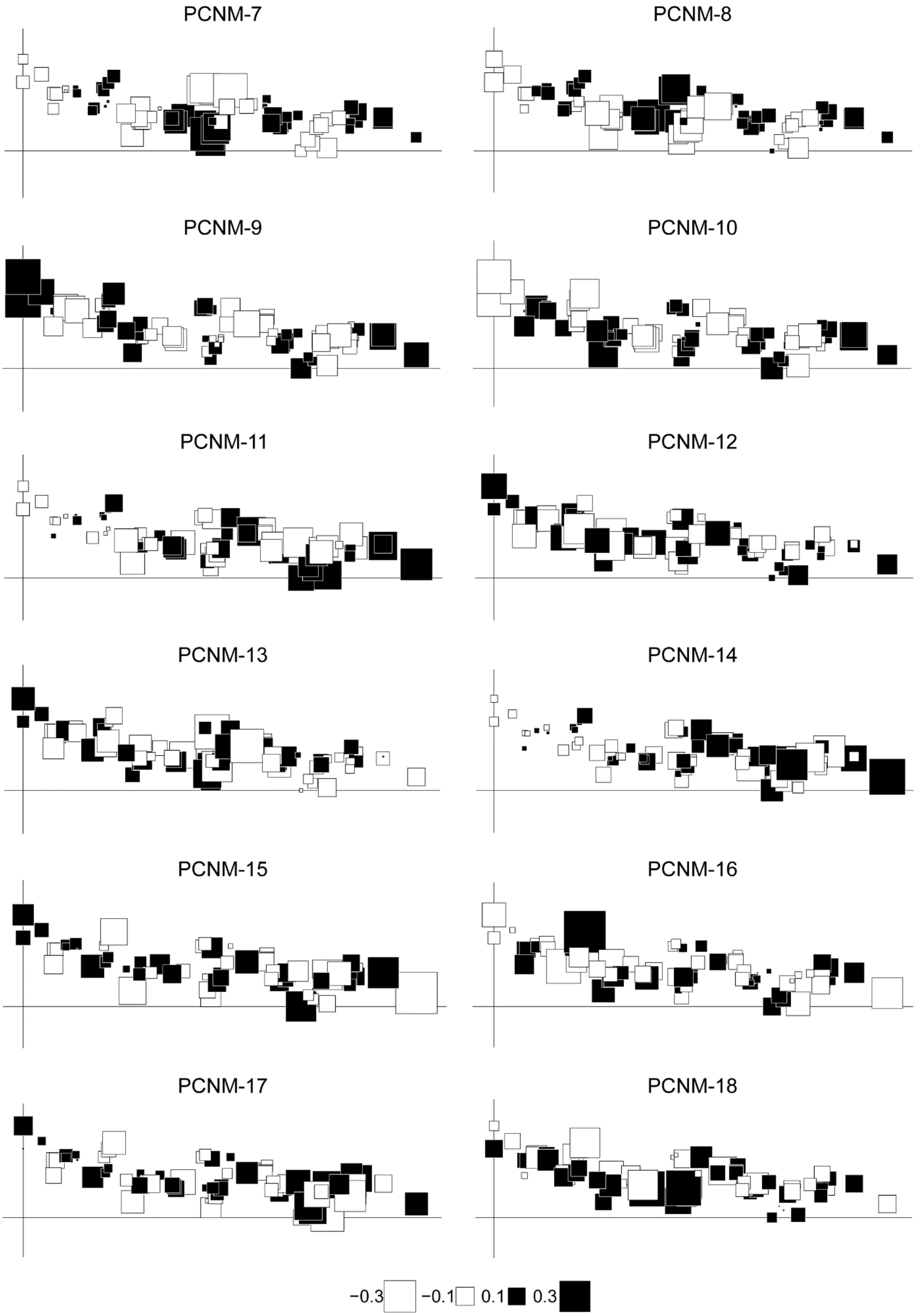

S1 Appendix (continued) Maps of PCNMs: medium-scale PCNMs (II), from PCNM-19 to PCNM-30.

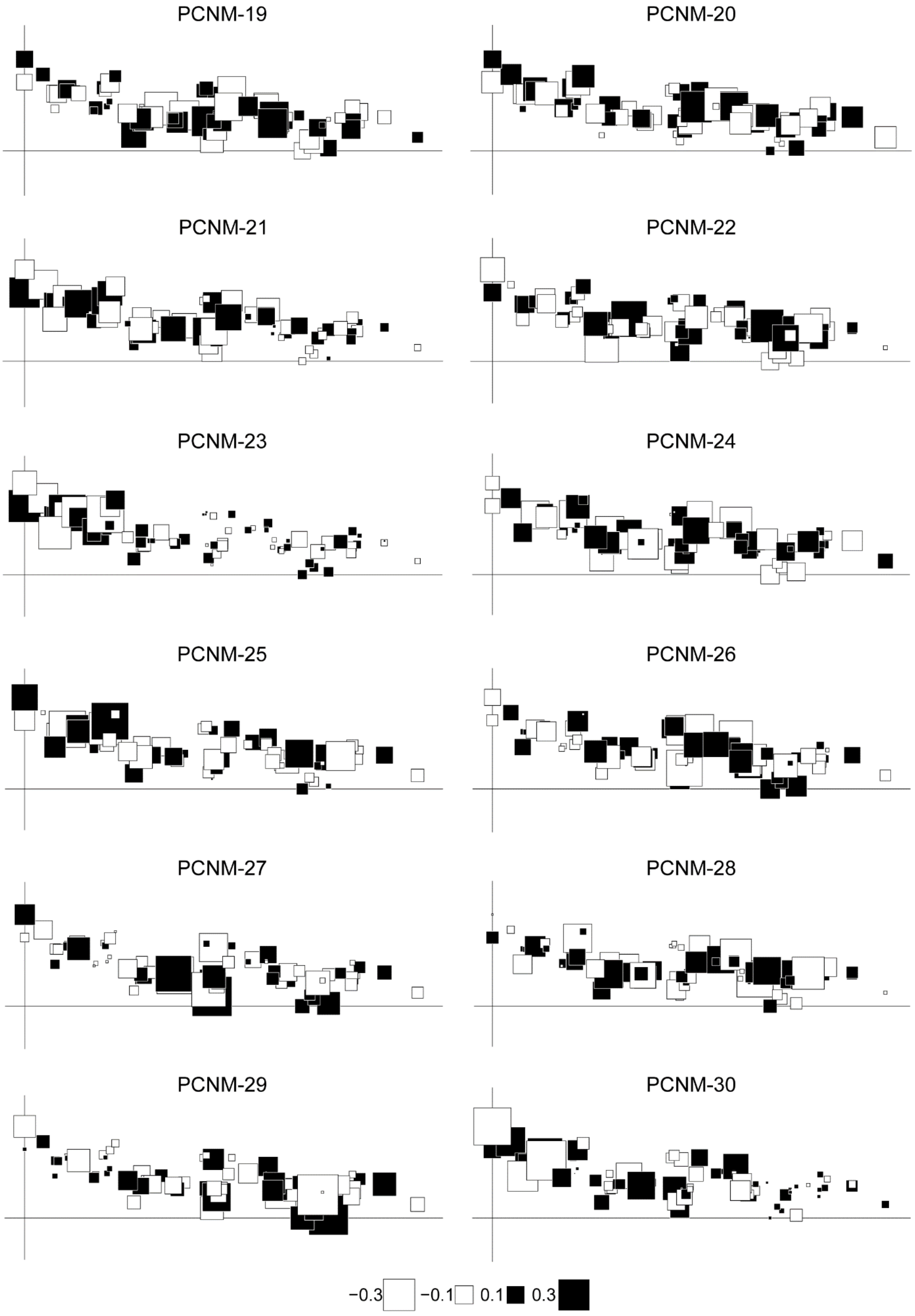

S1 Appendix (continued) Maps of PCNMs: small-scale PCNMs (I), from PCNM-31 to PCNM-40.

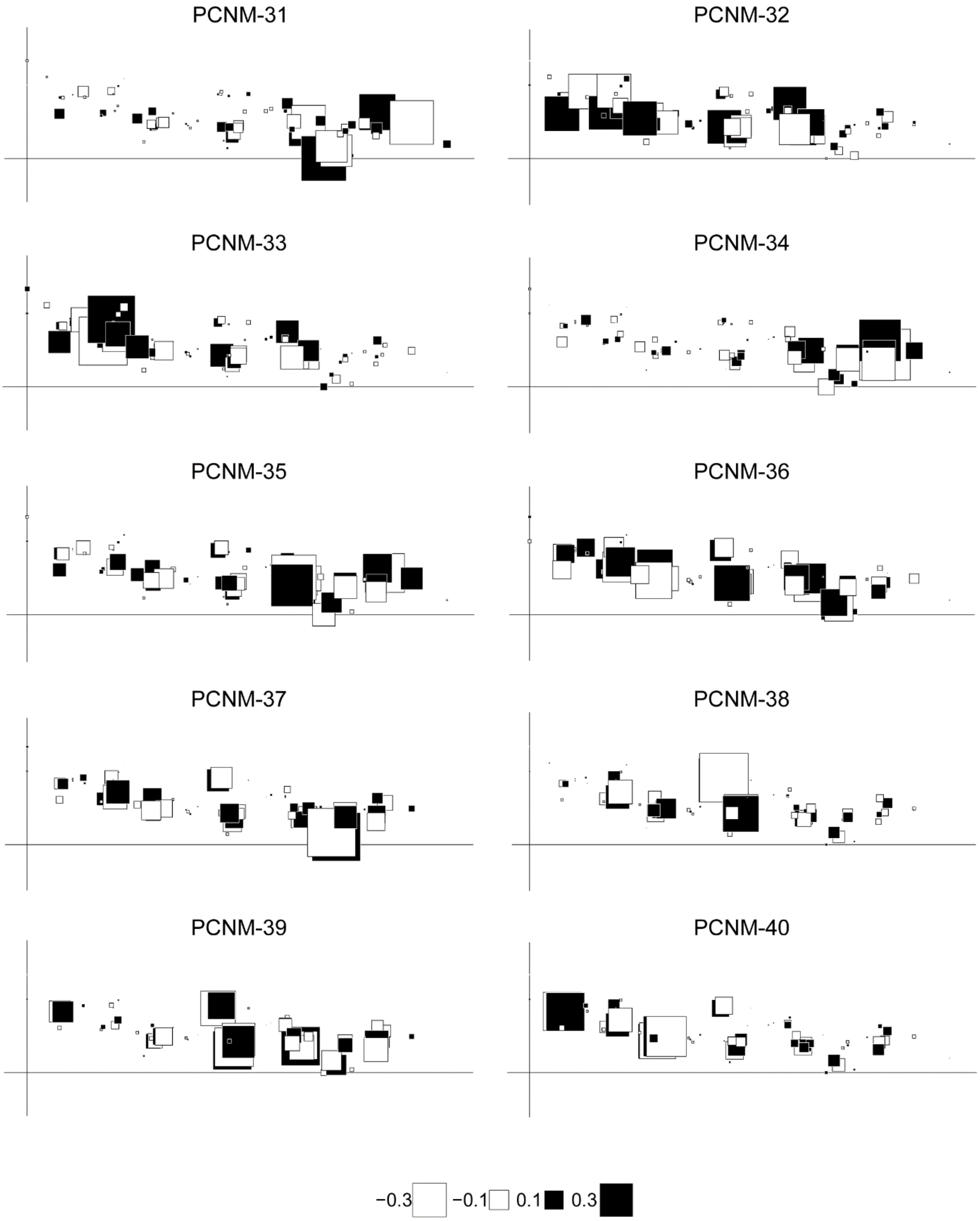

S1 Appendix (continued) Maps of PCNMs: small-scale PCNMs (II), from PCNM-41 to PCNM-45.

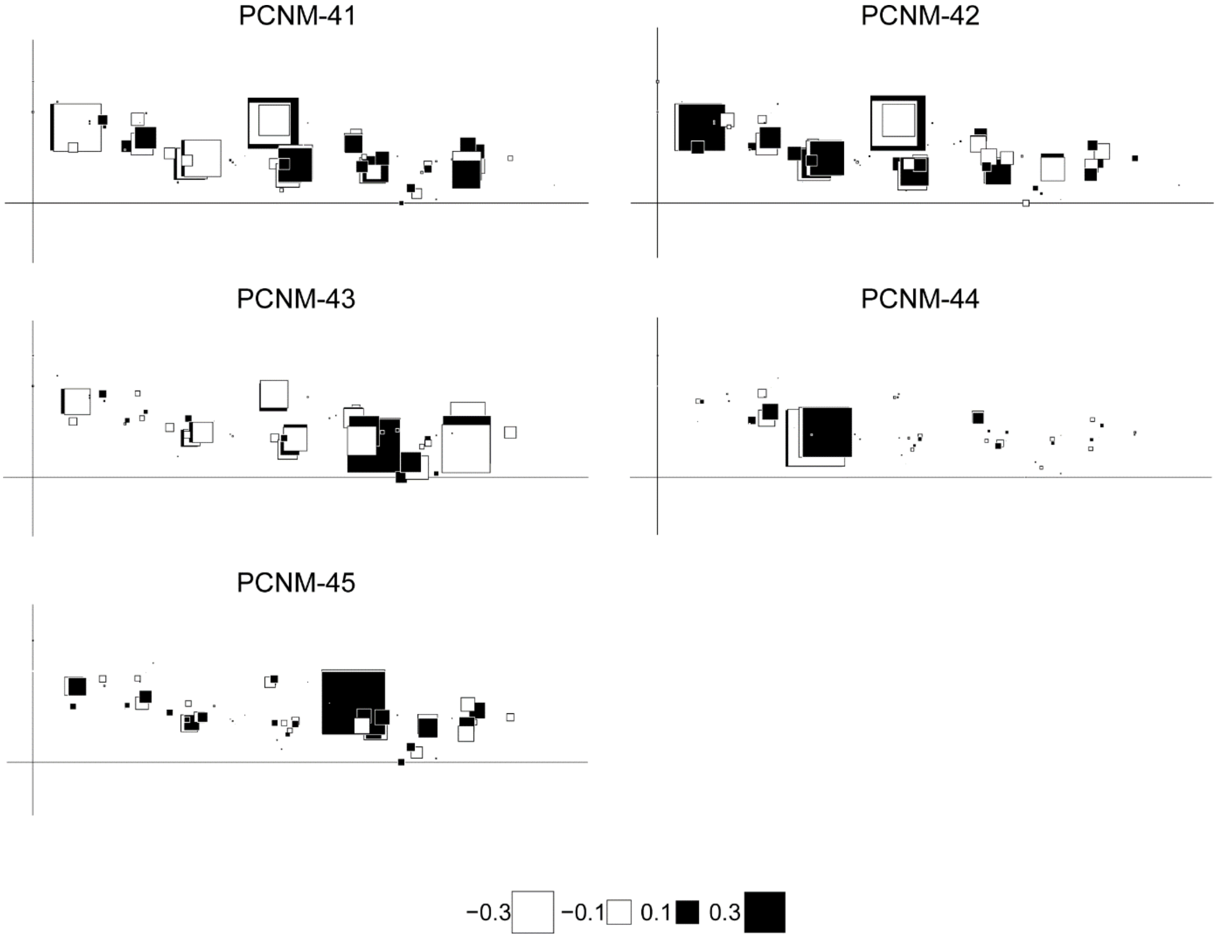

Supplement: S1 Appendix — (PDF) [file pone.0303864.s002.pdf]
