# Supplementary material for: Female proportion has a stronger influence on dispersal than body size in nematodes of mountain lakes
Source: PLoS One. 2024 May 17;19(5):e0303864. doi: 10.1371/journal.pone.0303864 (PMC11101049; doi:10.1371/journal.pone.0303864)

Supporting Information for

Female proportion has a stronger influence on dispersal than body size

in nematodes of mountain lakes

G. de Mendoza, B. Gansfort, J. Catalan & W. Traunspurger

S2 Appendix Maps of the presence (black squares) /absence (white squares) distribution of the 20 nematode species included in the statistical analyses (i.e., species present in at least four lakes and represented by at least six adults). Maps represent the Pyrenees in Cartesian space, to match those of the PCNMs (S1 Appendix). Moran's *I* spatial autocorrelation index is indicated in parentheses next to each species name (in boldface when significant, $P < 0.05$). Moran's *I* was built on a binary matrix (i.e., lakes are assumed as directly connected, or not, by the model), with a threshold distance equivalent to the maximum distance in a minimum spanning tree, keeping all lakes connected.

S2 Appendix Distribution of the 20 nematode species used in statistical analyses, and their Moran's *I*.

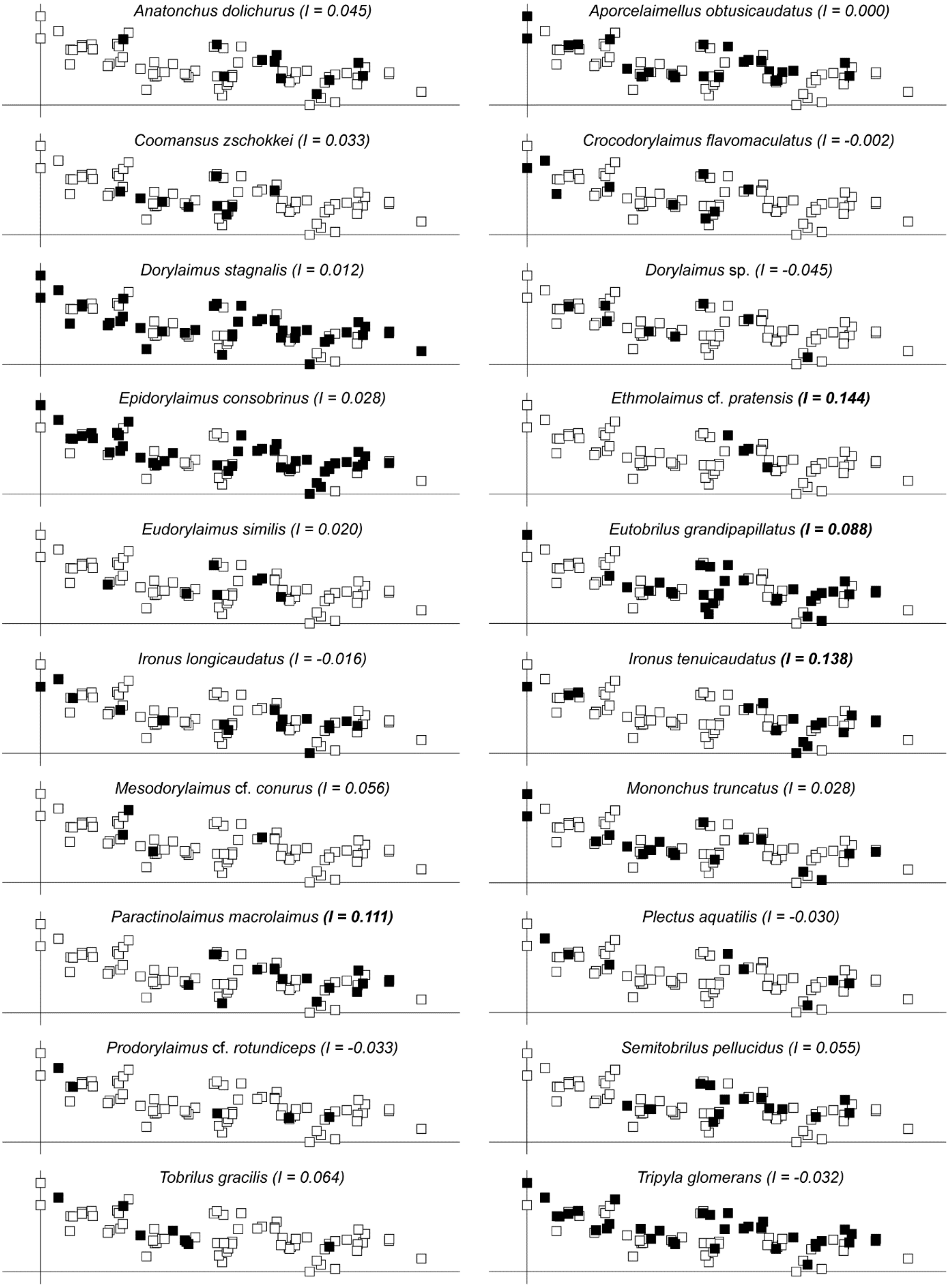

Supplement: S2 Appendix — (PDF) [file pone.0303864.s003.pdf]
