# Supplementary material for: Female proportion has a stronger influence on dispersal than body size in nematodes of mountain lakes
Source: PLoS One. 2024 May 17;19(5):e0303864. doi: 10.1371/journal.pone.0303864 (PMC11101049; doi:10.1371/journal.pone.0303864)
Supplement: S1 Table — (PDF) [file pone.0303864.s004.pdf]

Supporting Information for

Female proportion has a stronger influence on dispersal than body size

in nematodes of mountain lakes

G. de Mendoza, B. Gansfort, J. Catalan & W. Traunspurger

S1 Table Spearman's *rho* (and associated *P*-values in parenthesis), between the abundance of nematode species and the adjusted deviance (adj-D^2) accounted for by the different subsets of spatial and environmental variables considered in this study (data from Table 3 in the main manuscript), as well as between nematode species abundance and their Moran's *I* (data from Table 1 in the main manuscript). Abundance is considered as average values per species, first excluding zeros and then including all values, and also as maximum abundance per species. All Spearman's *rho* values obtained are not significant ($P > 0.05$).

	**Average abundance (without zeros)**	**Average abundance (with zeros)**	**Maximum abundance**
adj-D^2 large-scale PCNMs	−0.009 (*P* = 0.971)	0.029 (*P* = 0.903)	0.023 (*P* = 0.924)
adj-D^2 medium-scale PCNMs	−0.095 (*P* = 0.689)	−0.341 (*P* = 0.141)	−0.070 (*P* = 0.770)
adj-D^2 small-scale PCNMs	−0.303 (*P* = 0.194)	−0.291 (*P* = 0.212)	−0.245 (*P* = 0.298)
adj-D^2 env. variables	0.116 (*P* = 0.625)	0.006 (*P* = 0.980)	0.198 (*P* = 0.402)
Moran's *I*	−0.043 (*P* = 0.858)	−0.064 (*P* = 0.789)	−0.094 (*P* = 0.693)
