# Supplementary material for: Female proportion has a stronger influence on dispersal than body size in nematodes of mountain lakes
Source: PLoS One. 2024 May 17;19(5):e0303864. doi: 10.1371/journal.pone.0303864 (PMC11101049; doi:10.1371/journal.pone.0303864)
Supplement: S2 Table — (PDF) [file pone.0303864.s005.pdf]

# Supporting Information for

*Female proportion has a stronger influence on dispersal than body size*

*in nematodes of mountain lakes*

G. de Mendoza, B. Gansfort, J. Catalan & W. Traunspurger

**S2 Table (next two pages)** Female proportion and body size data per lake, with geographic coordinates. Female proportion was estimated either as average between species at each lake (only species with at least six adults in a given lake were considered), or as values weighed by species abundance at each lake (only lakes with at least 6 adults, independently of the species, were considered). Alt, altitude (m a.s.l.); Long, longitude E; Lat, latitude N; PF avg, proportion of females (average between species); PF w, proportion of females (weighed by species abundance); rel BS avg, relative body size (average between species); rel BS w, relative body size (weighed by species abundance). Body size values are relative to the largest value per lake. Female data are not available (n/a) if no adults were collected, or not considered (n/c) if the criteria for selection above described were not met. Lakes are listed by increasing altitude.

**S2 Table**

| Lake name | Alt | Long | Lat | PF avg | PF w | rel BS avg | rel BS w |
|---|---|---|---|---|---|---|---|
| Llebreta | 1620 | 0.89031 | 42.55083 | n/c | n/c | 0.077011 | 0.053385 |
| Compte | 1726 | 1.79306 | 42.63366 | 0.682 | 0.720 | 0.218348 | 0.176060 |
| Estom | 1804 | −0.09853 | 42.80752 | n/c | n/c | 0.253602 | 0.207177 |
| Basa de la Mora | 1908 | 0.32771 | 42.54526 | n/c | n/c | 0.557950 | 0.386777 |
| Bleu de Rabassoles | 1920 | 1.97274 | 42.70038 | 0.721 | 0.631 | 0.467168 | 0.375665 |
| Rond | 1929 | 1.06450 | 42.79440 | 0.793 | 0.703 | 0.193070 | 0.205205 |
| Laurenti | 1936 | 2.02582 | 42.67525 | n/c | n/c | 0.329402 | 0.186914 |
| Ormiélas | 1974 | −0.35562 | 42.88380 | n/c | 0.846 | 0.174806 | 0.085558 |
| Llong | 2000 | 0.95063 | 42.57431 | n/c | n/c | 0.171448 | 0.090843 |
| Montagnon | 2003 | −0.49387 | 42.96846 | 0.762 | 0.723 | 0.257555 | 0.328652 |
| Estelat | 2021 | 2.21351 | 42.64632 | 0.511 | 0.500 | 0.333597 | 0.201610 |
| Puis | 2056 | 0.70760 | 42.65542 | 0.472 | 0.472 | 0.557950 | 0.386777 |
| Asnos | 2060 | −0.26629 | 42.69180 | n/c | n/c | 0.299120 | 0.117642 |
| Aygue Longue | 2076 | 1.88263 | 42.64189 | 0.972 | 0.979 | 0.153921 | 0.035198 |
| Bersau | 2077 | −0.49454 | 42.84062 | 0.798 | 0.900 | 0.211866 | 0.210952 |
| Negre | 2083 | 2.21141 | 42.63592 | 0.692 | 0.730 | 0.261890 | 0.190560 |
| Les Laquettes 1 | 2085 | 0.14806 | 42.83592 | 1.000 | 0.923 | 0.737090 | 0.610633 |
| Aubé | 2094 | 1.33801 | 42.74549 | n/c | n/c | 0.683539 | 0.327483 |
| Romedo de Dalt | 2110 | 1.32465 | 42.70601 | n/c | 0.800 | 0.591967 | 1.000000 |
| Senó | 2130 | 1.32291 | 42.71203 | n/c | 0.846 | 0.494581 | 0.186154 |
| Long de Liat | 2140 | 0.87398 | 42.80655 | n/c | 0.750 | 0.672872 | 0.373770 |
| L'Estagnol | 2164 | 2.46276 | 42.53361 | n/a | n/a | 0.557950 | 0.386777 |
| Gerber | 2170 | 0.99471 | 42.63065 | n/c | n/c | 0.335841 | 0.232809 |
| Siscar | 2187 | 1.74718 | 42.60140 | 0.824 | 0.788 | 0.404777 | 0.194054 |
| Plan | 2188 | 0.93070 | 42.62248 | n/a | n/a | 1.000000 | 0.918390 |
| Pixón | 2199 | 0.37986 | 42.63682 | n/c | n/c | 0.055846 | 0.044458 |
| Airoto | 2210 | 1.03922 | 42.70281 | 0.375 | 0.583 | 0.246436 | 0.326793 |
| Arratille | 2247 | −0.17364 | 42.80158 | n/c | n/c | 0.406350 | 0.316717 |
| Malniu | 2250 | 1.79238 | 42.47378 | n/c | n/c | 0.119852 | 0.081669 |
| Gros de Camporrells | 2255 | 2.00788 | 42.62583 | 1.000 | 0.945 | 0.328106 | 0.223780 |
| Inferior de la Gallina | 2270 | 1.18763 | 42.70618 | 0.870 | 0.800 | 0.182094 | 0.167713 |
| Mariola | 2276 | 1.22434 | 42.71737 | 0.718 | 0.775 | 0.370849 | 0.156512 |
| Nere de Güèrri | 2280 | 0.85029 | 42.79334 | n/c | 1.000 | 0.237256 | 0.227986 |
| Port Bielh | 2290 | 0.18846 | 42.87417 | n/c | n/c | 0.188448 | 0.110527 |
| Mes amunt de Tristaina | 2300 | 1.48741 | 42.64685 | n/c | n/c | 0.229887 | 0.132933 |
| Arnales | 2305 | −0.24231 | 42.77503 | n/c | 0.833 | 0.151473 | 0.084644 |
| Trebens | 2306 | 1.96255 | 42.57780 | 0.625 | 0.700 | 0.271325 | 0.185883 |

**S2 Table (continued)**

| Lake name | Alt | Long | Lat | PF avg | PF w | rel BS avg | rel BS w |
|---|---|---|---|---|---|---|---|
| Pica Palòmera | 2308 | 0.86878 | 42.79377 | 0.810 | 0.833 | 0.307344 | 0.302184 |
| Sotllo | 2346 | 1.38445 | 42.65200 | 1.000 | 1.000 | 0.236338 | 0.222814 |
| Blaou | 2350 | 1.57264 | 42.65500 | 0.981 | 0.973 | 0.224772 | 0.055923 |
| Gran de la Pera | 2350 | 1.59509 | 42.45818 | n/c | n/c | 0.222364 | 0.149751 |
| Albe | 2355 | 1.74514 | 42.61835 | 0.679 | 0.573 | 0.179778 | 0.147064 |
| Sen | 2360 | 0.39312 | 42.62148 | n/c | n/c | 0.254749 | 0.176595 |
| Aixeus | 2370 | 1.37180 | 42.61098 | n/c | 0.857 | 0.408626 | 0.236521 |
| Montoliu | 2375 | 0.92614 | 42.78467 | n/c | n/c | 0.211654 | 0.084298 |
| Barroude Inf. | 2377 | 0.14478 | 42.73264 | 0.701 | 0.786 | 0.169181 | 0.160558 |
| Urdiceto | 2378 | 0.28160 | 42.66672 | 0.947 | 0.966 | 0.206681 | 0.294681 |
| Canals Roges | 2410 | 1.71180 | 42.58673 | n/c | 1.000 | 0.236984 | 0.107957 |
| Eriste | 2411 | 0.46808 | 42.64646 | 1.000 | 1.000 | 0.142394 | 0.035796 |
| Monges | 2418 | 0.87701 | 42.62301 | 0.869 | 0.848 | 0.166560 | 0.085555 |
| Angonella de Mes Amunt | 2440 | 1.48138 | 42.61015 | n/c | n/c | 0.557950 | 0.386777 |
| Montmalús | 2440 | 1.68263 | 42.49832 | 1.000 | 0.778 | 0.287317 | 0.095612 |
| Illa | 2452 | 0.99348 | 42.61836 | n/c | 0.909 | 0.200251 | 0.135486 |
| Llosás | 2480 | 0.65483 | 42.61766 | n/c | 1.000 | 0.384037 | 0.234311 |
| Baiau Superior | 2480 | 1.43188 | 42.59627 | n/c | n/c | 0.171108 | 0.099287 |
| Gran del Pessó | 2493 | 0.91563 | 42.51264 | 0.496 | 0.504 | 0.315866 | 0.384489 |
| Gelat Bergús | 2493 | 0.96331 | 42.59106 | n/c | 1.000 | 0.136937 | 0.059053 |
| Col d'Arratille | 2501 | −0.17274 | 42.79104 | 0.681 | 0.481 | 0.491904 | 0.361290 |
| Blau | 2531 | 1.96708 | 42.61554 | 1.000 | 1.000 | 0.032386 | 0.027011 |
| La Munia Sup. | 2537 | 0.12499 | 42.70615 | 0.758 | 0.623 | 0.359010 | 0.377759 |
| Posets | 2550 | 0.44940 | 42.64681 | 0.971 | 0.958 | 0.279969 | 0.065442 |
| Ensangents Sup. | 2550 | 1.64923 | 42.52134 | 1.000 | 0.983 | 0.536478 | 0.638725 |
| Glacé | 2571 | −0.08845 | 42.77830 | n/a | n/a | 0.039271 | 0.027223 |
| Helado de Marboré | 2592 | 0.04104 | 42.69659 | 1.000 | 0.981 | 0.239551 | 0.083213 |
| Negre | 2627 | 1.43826 | 42.58913 | n/c | 0.769 | 0.278040 | 0.177523 |
| Bachimala Sup. | 2630 | 0.38761 | 42.70440 | n/c | n/c | 0.453653 | 0.314478 |
| Forcat Inf. | 2631 | 1.44883 | 42.60074 | 0.875 | 0.900 | 0.135917 | 0.083454 |
| Tourrat | 2636 | 0.09966 | 42.80998 | n/c | n/c | 0.039271 | 0.027223 |
| Cregüeña | 2640 | 0.62530 | 42.63867 | 0.941 | 0.929 | 0.277615 | 0.248770 |
| Lliterola | 2734 | 0.53380 | 42.69367 | 0.862 | 0.833 | 0.155468 | 0.081296 |
| Coronas | 2740 | 0.63848 | 42.62997 | 0.618 | 0.684 | 0.188667 | 0.135395 |
| Pondiellos Sup. | 2745 | −0.26312 | 42.77699 | 0.714 | 0.739 | 0.246462 | 0.296524 |
| Chelau Sup. | 2805 | 0.40667 | 42.62419 | 1.000 | 1.000 | 0.082621 | 0.045254 |
| Cap Long | 2845 | 0.11305 | 42.79510 | 0.590 | 0.594 | 0.561520 | 0.745093 |
| Helado del Mte. Perdido | 2990 | 0.02771 | 42.68213 | n/c | n/c | 0.364529 | 0.208002 |
